# Interactions between extracellular vesicles and viruses: lessons learned across species and kingdoms

**DOI:** 10.1093/femsre/fuag028

**Published:** 2026-06-23

**Authors:** Kyra Defourny, Paola Xhelili, İkbal Agah Ince, Fabrizio Cillo, Karolina Sapoń, Tadeusz Janas, Guy Smagghe, Erinda Lika, Amy H Buck, Esther Nolte-’t Hoen, Kriton Kalantidis, Konstantina Katsarou

**Affiliations:** VIB Center for Inflammation Research, VIB, 9052 Ghent, Belgium; Department of Biomedical Molecular Biology, Ghent University, 9000 Ghent, Belgium; Department of Biomolecular Health Sciences, Division of Infectious Diseases and Immunology, Faculty of Veterinary Medicine, Utrecht University, 3584 CL Utrecht, The Netherlands; Department of Agronomic Sciences, Faculty of Agriculture and Environment, Agricultural University of Tirana, Tirana 1025, Albania; Centre de Biologie Structurale (CBS), Université Montpellier, CNRS, INSERM, 34090 Montpellier, France; Institute for Sustainable Plant Protection, National Research Council of Italy (CNR), 70126 Bari, Italy; Institute of Biology, University of Opole, 45-032 Opole, Poland; Institute of Biology, University of Opole, 45-032 Opole, Poland; Institute of Entomology, Guizhou University, Guiyang, 550025, China; Department of Biology, Vrije Universiteit Brussels (VUB), 1050 Brussels, Belgium; Faculty of Veterinary Medicine, Agricultural University of Tirana, Tirana 1025, Albania; Institute of Immunology and Infection Research, School of Biological Sciences, University of Edinburgh, Edinburgh EH9 3FL, United Kingdom; Department of Biomolecular Health Sciences, Division of Infectious Diseases and Immunology, Faculty of Veterinary Medicine, Utrecht University, 3584 CL Utrecht, The Netherlands; Department of Biology, University of Crete, Voutes University Campus, 71409 Heraklion, Crete, Greece; Institute of Molecular Biology and Biotechnology, Foundation for Research and Technology-Hellas, 71110 Heraklion, Crete, Greece; Department of Biology, University of Crete, Voutes University Campus, 71409 Heraklion, Crete, Greece; Institute of Molecular Biology and Biotechnology, Foundation for Research and Technology-Hellas, 71110 Heraklion, Crete, Greece

**Keywords:** extracellular vesicles (EVs), exosomes, microvesicles, apoptotic bodies, virus–host interactions, infection

## Abstract

Extracellular vesicles (EVs) are membranous nanoparticles released by cells that help shape the extracellular environment, remove cellular waste, and mediate cell-to-cell communication. Their release is ubiquitous across kingdoms, species, and cell types, highlighting their functional importance. Nearly as evolutionarily widespread and heterogeneous is the release of viruses, which have evolved to co-opt the host’s cellular machinery to facilitate their replication and spread within all branches of life. Nearly all viruses, enveloped or not, repurpose EVs to modulate infection dynamics, while EVs also play a crucial role in the host’s response to infection. This review explores the interplay between EVs and viruses across the phylogenetic diversity of virus species. We urge virologists and EV biologists to look beyond a single infection model and learn from the unique concepts and shared commonalities observed between close, as well as distantly related viruses, whether they infect mammals, vertebrates, insects, plants, bacteria, or more. To facilitate these efforts, we provide a comprehensive, taxonomical overview of the current knowledge regarding DNA and RNA virus families, and discuss recurring motifs in EV release and function during infection.

## Teaser

A closer look at the full diversity of interactions between viruses and host-derived extracellular vesicles, across the entire viral kingdom and its related host range. Created in BioRender. Defourny, K. (2026) https://BioRender.com/1n6z8wu.

## Introduction

Extracellular vesicles (EVs) are cell-derived, membrane-enclosed nanoparticles that carry a large variety of cargo molecules within and across organisms, including RNA, DNA, proteins, and lipids. EVs are heterogeneous in both their molecular compositions and biogenesis pathways, and can derive from different subcellular membranes. Based on their origin, EVs are often classified into subgroups. Examples in the mammalian field include exosomes (derived from endosomal compartments), microvesicles or ectosomes (derived from the plasma membrane, PM), and apoptotic bodies (produced during fragmentation of apoptotic cells, ApoBDs), whereas EVs originating from bacteria are grouped as outer membrane vesicles (OMV), inner membrane vesicles, or outer–inner-membrane vesicles. However, these different EV subsets often cannot be distinguished with certainty due to overlapping sizes, protein and lipid profiles, and different classification criteria are used depending on the studied organism. For example, the biogenesis of plant EVs remains elusive and is subject to its own developing classification system, but likely shares (some) common aspects with EV subsets recognized in other organisms. An alternative practice is to report on EVs based on their isolation method. This is because experiment design often restricts analysis to a certain EV subpopulation (such as those with a certain sedimentation coefficient, size, or molecular composition), creating a selective bias within the obtained results (Doyle and Wang 2019). However, this approach also complicates extrapolation between studies and organisms. Hence, a more generic distinction of small (30–200 nm, sEVs) versus large (200 nm—several µm, lEVs) EVs is generally recommended (see MISEV guidelines; Welsh et al. 2024).

The production of EVs is highly conserved and has been observed for nearly all cells studied to date, including those long believed incapable of EV release due to the presence of a surrounding plant, fungal, or bacterial cell wall (Woith et al. 2019, Stotz et al. 2022). In fact, organisms across all kingdoms are currently recognized to employ EVs to (1) remove unwanted cellular cargo, (2) modulate the extracellular matrix, (3) trigger receptor-mediated signaling, or (4) deliver cargo directly into the cytoplasm of a recipient cell, among other (context-dependent) functions (Niel van et al. 2018, Woith et al. 2019, Buck and Nolte-’t Hoen 2024). EVs can even directly transfer signals across species and kingdom boundaries, as evidenced by observations of EV-mediated cross-talk between fungi and plants, as well as mammalian hosts and their bacterial, protozoan, and invertebrate pathogens, further supporting their role as a ‘universal cell language’ (Wu et al. 2019b, Stotz et al. 2022, Zhang et al. 2024a). The conserved nature of EV biology prompts the need for a collective approach to EV research, as opposed to current, often compartmentalized efforts, especially in the frame of EVs and viruses. First, a growing body of literature describes overlapping anti- or proviral roles for EVs in infection settings that span the full viral kingdom, whether it concerns DNA or RNA, enveloped or naked viruses. Secondly, seemingly diverse infection settings typically share the same technical challenges that accompany the study of EVs during infection (summarized in Box 1). Hence, there are clear arguments urging the exchange of both practices and discoveries across apparent biological confines, and promoting the integration of the field as a whole.

**Figure 1:**
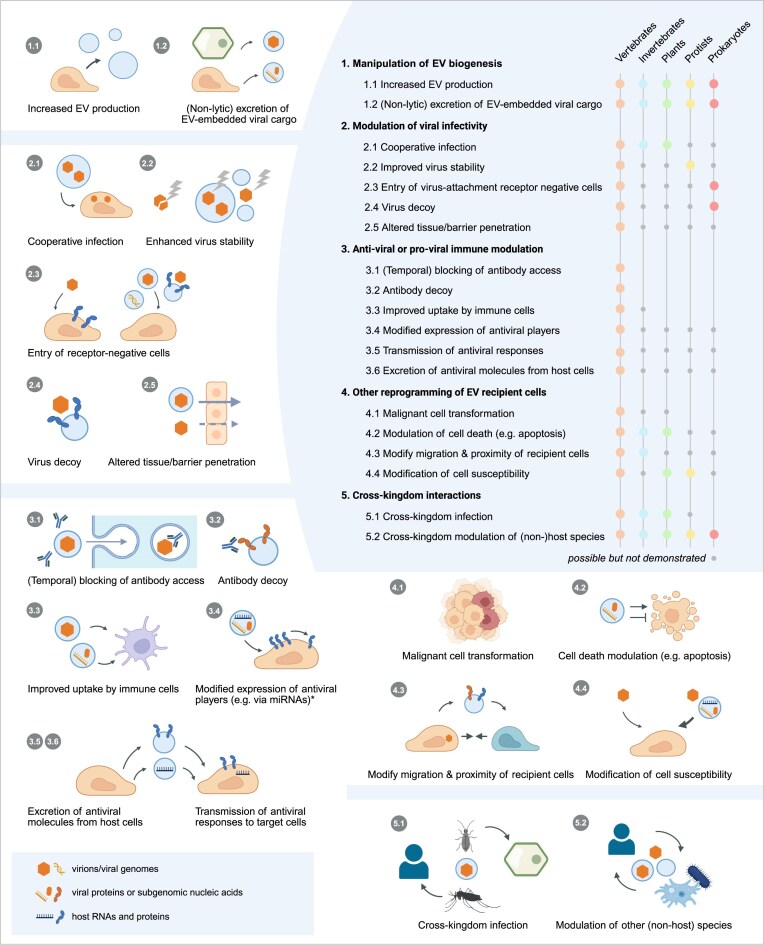
Recurring functions of EVs in infection across the full diversity of viruses. Schematic depiction of functions that have been attributed to EVs in the context of at least two different virus families. Grey dots denote functions that have not yet been attributed to the EVs in question, but for which preliminary indications suggest an EV–mediated role is possible and may be confirmed in future studies. Created in BioRender (Defourny, K. 2026; https://BioRender.com/x1d689q).

**Figure 2:**
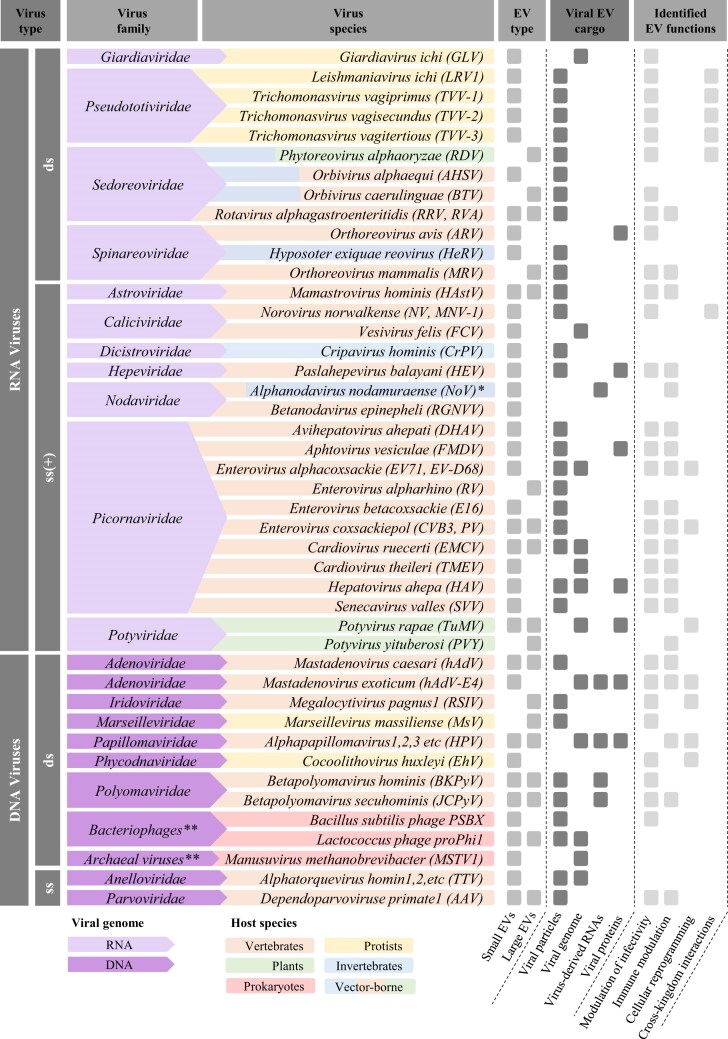
The packaging of viral cargo within EVs across nonenveloped (naked) virus species. Listed is the type of viral cargo identified within EVs for each of the indicated viruses. Viruses are identified by their formal species classification, as well as the abbreviation of the common strain name in brackets ‘‘()’’. Analysis of small vs. large EV subsets is indicated using a 200 nm cut-off in accordance with MISEV guidelines, or, when sizing information is not available, based on the pelleting of EVs at 10 K (lEVs) vs. 100 K (sEVs), respectively. The role of EVs is represented in four different columns, containing modulation of infectivity, immune modulation, cellular reprogramming, and cross-kingdom interactions. Listed host species refer to the species in which the virus replicates, including intermediary virus vectors, regardless of whether all host species have been studied in relation to EVs. (+) = positive sense, ds = double-stranded, and ss = single-stranded. *Not vector-borne but capable of replicating in diverse hosts. **Given the recent changes in the taxonomy of bacterial and archaeal viruses, we here refer to this subgroup and its species using the descriptive names as employed in the cited literature.

**Figure 3:**
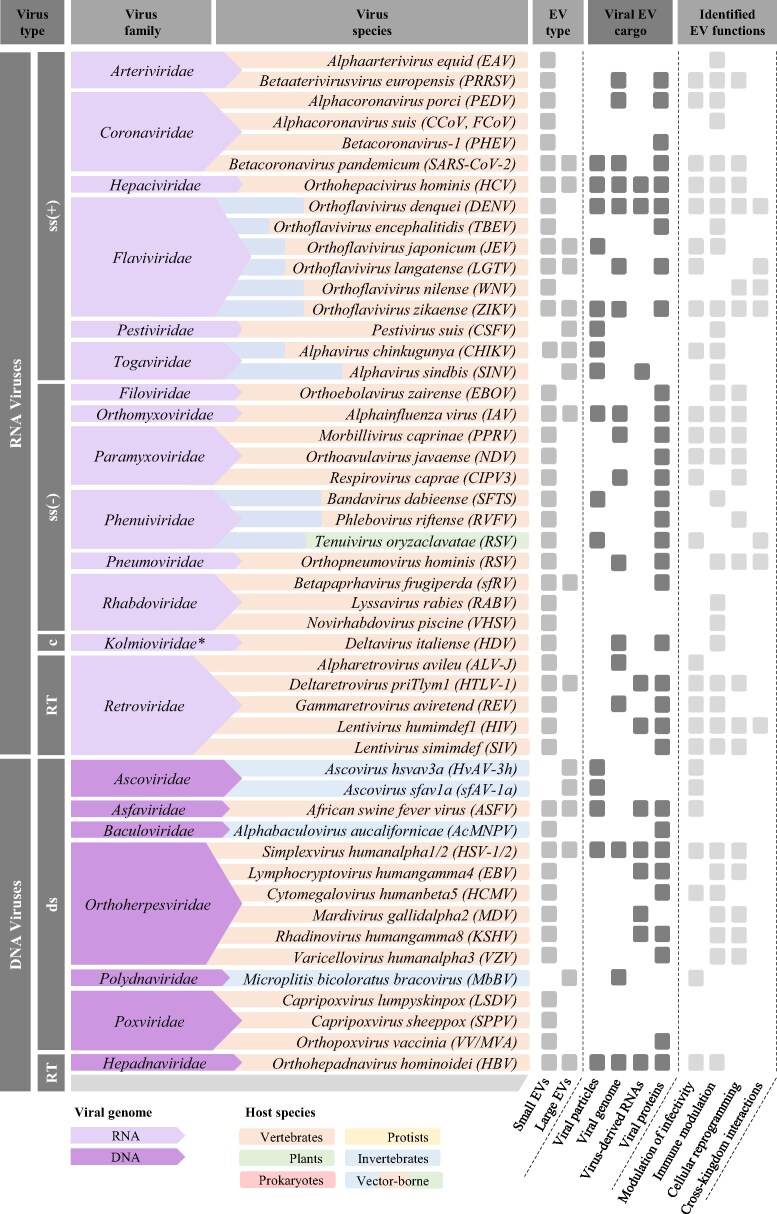
The packaging of viral cargo within EVs across enveloped virus species. Listed is the type of viral cargo identified within EVs for each of the indicated viruses. Viruses are identified by their formal species classification, as well as the abbreviation of the common strain name in brackets ‘‘()’’. Analysis of small vs. large EV subsets is indicated using a 200 nm cut-off in accordance with MISEV guidelines, or, when sizing information is not available, based on the pelleting of EVs at 10 K (lEVs) vs. 100 K (sEVs), respectively. The role of EVs is represented in four different columns, containing modulation of infectivity, immune modulation, cellular reprogramming, and cross-kingdom interactions. Listed host species refer to the species in which the virus replicates, including intermediary virus vectors, regardless of whether all host species have been studied in relation to EVs. (±) = positive or negative sense, c = circular, ds = double-stranded, and RT = reverse transcribing. *Employs the envelope of a helper virus.

In this review, we propose that due to the conserved nature of EV release, EVs are part of integral host cell processes that viruses have coevolved with. As a result, we predict recurring patterns of the use or modulation of EVs by distant virus species. To help uncover such patterns, we have examined publications on the full diversity of different virus families, covering both well-characterized and lesser-known cases, and discuss their proposed interactions with EVs. We present readers a conceptual overview of the functions attributed to EVs across multiple virus lineages, based on observations within or across host kingdoms (Fig. 1). In addition, we provide an encyclopedic overview of the current state-of-the-art in each individual arm of the viral kingdom, allowing an unbiased review of all studied viruses regardless of their overall representation in current literature. This overview spans RNA (Fig. 2) and DNA (Fig. 3) viruses grouped according to their taxonomic classification, as well as a separate discussion of the viruses infecting prokaryotes since their taxonomy is currently undergoing revision. Virus families have been further separated based on their classification as nonenveloped (naked) or enveloped viruses, in light of the structural and biogenesis similarities between enveloped viruses and EVs. By adopting this structure, we guide newcomers and experts in a narrative way through the recurring themes in EV–virus interactions, and refer readers to dedicated sections on relevant virus families for details and the underlying literature. Our goal is to foster knowledge exchange across infection models, whether they concern viruses infecting mammals, arthropods, plants, or other hosts, and to advance the field by promoting readers to consider the full diversity of findings with a unifying, EV-centric perspective in mind.

Box 1: Technical challenges related to the study of EVs during virus infection.

Isolation and purification of EVs involves techniques like ultracentrifugation, size-exclusion chromatography, density-gradient centrifugation, or antibody pull-down. However, each technique has its own disadvantages regarding recovery rate, co-isolation of different contaminants, time and resource investment, or the loss of specific EV subsets. These issues are exacerbated in virus infection studies. First, the induction of virus-induced cell death or stress can lead to the release of increased levels of contaminants, including those that may hamper EV isolation (e.g. DNA). Markers used to assess the purity of EVs, like ER or Golgi-proteins, may be unreliable due to virus-driven rearrangement of cellular membranes and often do not exclude all relevant contaminants (including soluble proteins). Correct data interpretation requires sufficient knowledge of the contaminants that can be expected with a given technique.

A second, even bigger challenge is separating virus particles from EVs, which varies across virus families. Viruses can (partially) overlap with EVs in size, sedimentation coefficient, precipitation tendency, and buoyant density, all common features used for EV isolation. The difficulty increases with enveloped viruses due to their morphological and compositional similarities to EVs, arising from their shared use of cellular membranes and budding pathways. This similarity complicates proper virus–EV separation, as viral proteins can be integrated in EVs and *vice versa*, causing affinity-based capture methods to inadvertently target EVs or virions with mixed composition. Additionally, viruses may bind to the surface of EVs, via direct binding to EV membrane components or to other molecules that become part of the EV protein corona, leading to co-isolation. As a result of these challenges, data interpretation may be complicated by contaminating virions. Hence, studies claiming to detect infectious EV subsets ought to be subject to rigorous controls.

EVs can carry a wide variety of viral material, including small RNAs, soluble or transmembrane proteins, replication complexes, defective viral genomes (DVGs), full viral genomes, and even entire capsids of naked and enveloped viruses. Even complete enveloped virions have the potential to end up within secondary EV membranes, depending on the site of viral envelope acquisition, and/or the complexity of the EVs in question [e.g. secreted multivesicular bodies (MVBs) or ApoBDs with internal vesicular structures]. This heterogeneity complicates discrimination of the type of cargo carried by EVs, an endeavour that is further challenged by the fact that the molecules detected in EV isolates may be packaged within the same EV particle or across multiple EV subsets. Hence, multiple assays are often required to distinguish the uptake of full viral particles from that of subviral components. Relevant analysis strategies to verify true EV packaging (as opposed to the detection of co-isolated contaminants), include electron microscopy, antibody/protease accessibility, analysis of the presence and ratio of different components expected in mature virions, as well as context-dependent readouts. However, given the technical and biological complexity, including the fact that often heterogeneous mixtures of particles are released, we recommend caution when interpreting the presented data of any study. Currently, there are no strict definitions that specify the boundaries between an EV containing virus material and a genuine virus particle, leading to field-specific practices when it comes to classifying particles as EVs, defective virions, alternative virus packaging forms, or virus-like particles (VLP; the latter of which typically lack viral genetic material and promote their own assembly). In this review, we use the broadest definition of EVs to facilitate knowledge transfer between fields.

## A conceptual overview of recurring patterns in EV release and function across viral lineages

### Recurring modulation of EV biogenesis pathways

Increased EV production is a common feature of viral infection (Fig. 1-1.1), emphasizing the importance of virus-induced modulation of EV biogenesis. Common motifs that viruses rely on to modify EV release or composition in the eukaryotic context include Rab GTPases, endosomal sorting complexes required for transport (ESCRT) components (e.g. Alix, TSG101 for vertebrate EVs), tetraspanins (e.g. mammalian tetraspanins CD9/CD63), or the modulation of lipid biosynthesis (particularly the processing of sphingomyelin into ceramide), as was shown for example for *Polyomaviridae, Sedoreoviridae, Caliciviridae, Paramyxoviridae, Pestiviridae, Hepaciviridae, Hepeviridae, Picornaviridae, Hepadnaviridae, Orthoherpesviridae, Adenoviridae*, and *Marseilleviridae*. These molecules suggest a heavy reliance of eukaryotic viruses on typical exosomal biogenesis pathways (explained in detail in Teng and Fussenegger 2020, Martin et al. 2024), especially for the spread of viral subunits or functional cellular components. However, it should be noted that this trend may be biased by the relative accessibility of chemical and genetic inhibition strategies targeting this pathway, the reliability of which [including neutral sphingomyelinase (nSMase)-inhibitors] urges caution in data interpretation. Across the full variety of virus species, all major EV biogenesis pathways (reviewed in Rojas and Regev-Rudzki 2024) have been implicated in the release of virus-induced EVs. In addition, viruses from diverse families appear to promote the use of unconventional routes for EV packaging, particularly those based on the autophagy pathway, as was clearly demonstrated for *Picornaviridae, Flaviviridae, Pestiviridae, Sedoreoviridae*, and *Polyomaviridae*, although increased levels of autophagic markers have also been detected in *Retroviridae, Orthoherpesviridae*, and *Orthomyxoviridae* EVs. These EVs can carry virions enclosed within membranes of autophagosomes or amphisomes, which are fusion compartments between autophagosomes and multivesicular bodies (MVBs). However, molecular details governing the switch between autophagic degradation and secretion remain largely unclear. Multiple different autophagy-based secretory pathways exist (Zubkova et al. 2024), but the large-scale intracellular membrane changes typical to infection complicate identifying those affected by viruses. Another complication is the reliance of many viruses on autophagosomal membranes for replication or maturation, which limits the use of typical molecular tools for inhibiting different steps of the autophagy pathway, and in turn has driven the identification of novel regulators of autophagic secretion relevant to infection (*Picornaviridae* and *Flaviviridae*). Overall, the modulation of EVs parallels other conserved cellular processes targeted during infection, in the sense that different viruses achieve the same goals through a staggering variety of molecular mechanisms. When studying these mechanisms, it is important to consider that EV release from different pathways is not mutually exclusive, and cargo may be rerouted upon pathway blockage. Likewise, while there is a deserved interest in mechanisms for selective cargo packaging, nonspecific incorporation of viral cargo into EVs may also be biologically relevant.

Although the vast majority of information on EV production derives from the vertebrate system, evidence on the viral modulation of EV production in other organisms is slowly accumulating. Studies on viruses within *Sedoreoviridae, Potyviridae*, and *Flaviviridae* point to the modulation of similar (MVB-based) EV biogenesis pathways in arthropods and plant hosts. Various other plant viruses have been reported to target conserved EV biogenesis proteins, including tetraspanins and ESCRT proteins, and are therefore prime candidates for follow-up studies into EV release (Barajas et al. 2009, Rubino et al. 2014, Zhu et al. 2022). Similarly, reports of multiple plant viruses in autophagosome-like structures in their plant or intermediary insect host have been described, suggesting that the use of this pathway for virus release is likely more conserved than currently indicated (Liang et al. 2023, Liu et al. 2023) EV release during virus-induced cell death in particular is a process that is likely conserved all the way from prokaryotes to higher organisms. The role of lytic forms of cell death in prokaryotic EV release is increasingly emphasized, especially in the context of infection, while the eukaryotic field displays a bias toward the study of EVs from virus-induced apoptotic-like cell death (*Togaviridae, Iridoviridae, Ascoviridae, Asfarviridae, Polydnaviridae, Papillomaviridae*, and *Orthomyxoviridae*, with potential relevance also to *Parvoviridae and Paramyxoviridae*). The lack of well-documented molecular biogenesis pathways and/or specific markers for several EV types complicates the analysis of EVs in most organisms. Even in the well-studied vertebrate systems, microvesicle formation remains only partially understood and is still understudied in infection contexts. Hence, more conserved mechanisms of EV modulation are likely to emerge pending a better insight into different EV biogenesis pathways.

### Recurring functional signatures of EVs during viral infections

#### Transport of viral cargo and the direct modulation of infectivity

The use of EVs for the excretion of viral cargo is a finding that has long been dismissed as cellular debris. To this day, its study is still complicated by a lack of clear definitions separating EVs from virus-like or subviral particles, as well as alternate virus packaging forms (e.g. *Hepadnaviridae* and *Poxviridae*, see also Box 1). Nevertheless, the detection of viral cargo in EV isolates is considered the most widespread and consistent observation of EV–virus interplay (Fig. 1-1.2). Association of virus-derived molecules with EVs can drastically alter EV function and can even affect EV targeting, as viral proteins incorporated in the EV membrane or bound to EVs after release modify cellular uptake (demonstrated for *Hepadnaviridae, Orthomyxoviridae*, and *Bacteriophages*). The type of viral cargo found in EV isolates differs across infection settings, as can be seen in Figs 2 and 3, which summarize key findings for individual RNA and DNA virus species studied in relation to EVs. Among these, special attention is warranted to cases in which infectious material is packaged in the form of whole viral genomes or entire virions. By packaging infectious viral material in the EV lumen, EVs provide a new release strategy and transmission route within or between hosts. This is taken to extremes in nonenveloped virus species that appear to rely predominantly on membrane budding for excretion, raising questions on whether these viruses can still be considered strictly nonenveloped, and whether the (temporarily) acquired EV membranes should be considered an integral part of the virions (see *Sedoreoviridae, Spinareoviridae, Hepeviridae*, and *Picornaviridae*). For enveloped viruses, virus particles can even bud from the surface of released EVs, a phenomenon that may benefit the virus as the sequential use of EV-mediated and typical virus release pathways can enable prolonged virus production (proposed for *Ascoviridae* and *Orthomyxoviridae*).

Co-isolation of virions with EVs complicates the identification of EV-mediated virus release, an issue that is aggravated by the ability of EVs to bind virions encountered in the environment via surface interactions (see *Polyomaviridae, Picornaviridae*, and *Adenoviridae*). This binding is facilitated by the presence of many known viral receptors on EVs, including tetraspanin molecules (the most commonly used marker for EVs and receptor for *Hepaciviridae*, among others). The lack of rigorous controls confirming true EV packaging of viral cargo remains the number one criticism of published works. Yet, complete separation may not be necessary to address the properties of free virions versus those of EVs, as virions may be considered part of the functional EV protein corona (Heidarzadeh et al. 2023). In fact, both by binding virions to their surface and incorporating them into the EV lumen, EVs may affect the infectivity of viruses in several ways. First, observations of multiple virions in a single EV for both enveloped and naked viruses suggest cooperative infection is a key consequence of EV-mediated virus release (Fig. 1-2.1), a feature potentially also achieved by multiple virions binding to the EV surface. Interestingly, some enveloped virus species are historically described to produce diverse morphological forms of virus particles, including those carrying numerous capsids, though whether these structures qualify as EVs remains unclear (e.g. *Marseilleviridae* and *Baculoviridae*). The clustering of virus particles increases the number of virions entering per cell, also known as the multiplicity of infection (MOI), boosting replication efficiency according to both mathematical models and empirical evidence (Andreu-Moreno et al. 2020). However, when sustained or excessively high, elevated MOI can also promote the propagation of defective interfering virus particles. Currently, it remains unclear whether cooperative infection contributes to the enhanced infectivity of EV-associated viruses observed in some families (e.g. *Togaviridae, Flaviviridae, Picornaviridae*, and *Marseilleviridae*), or if other EV properties are responsible. Similarly, it is still unresolved whether increased cooperation between damaged virus particles explains the improved environmental stability of virions enclosed within or co-incubated with EVs (Fig. 1-2.2) (e.g. *Phycodnaviridae, Marseilleviridae, Caliciviridae*), rather than alternative mechanisms such as shielding. Advanced microscopy techniques and barcoded viruses may contribute to the investigation of the fate of jointly delivered viral genomes (Boersma et al. 2020, Smith et al. 2024), and the potential of EVs to promote cooperativity and/or recombination between quasispecies, as done in the context of *Spinareoviridae*.

Another key consequence of the association of viruses with EVs is the potential for virus internalization by cells that lack typical entry receptors (Fig. 1-2.3) (for example *Asfarviridae, Arteriviridae, Coronaviridae, Orthoherpesviridae*, and *Hepaciviridae*). Among others, this is because EVs can transport essential host receptors to target cells, or can facilitate virus–receptor-independent endocytosis by relying on other molecular motifs on the EV surface (shown for *Polyomaviridae, Adenoviridae, Coronaviridae, Picornaviridae, Bacteriophages*, and more). This same phenomenon also enables virus uptake independent of typical capsid maturation requirements (e.g. *Astroviridae*). Phosphatidylserine lipids (PS) exposed by EVs are the most commonly identified motif promoting the uptake of EV-enclosed viruses (e.g. *Asfarviridae, Hepeviridae*, and *Picornaviridae*), a process reminiscent of apoptotic mimicry employed by many enveloped virus species. At the same time, this abundance of PS-lipids enables EVs to compete with the uptake of free virions of numerous enveloped virus species relying on this motif for uptake (e.g. *Flaviviridae* and *Togaviridae*). A similar inhibitory effect has been shown for EVs carrying protein (e.g. *Coronaviridae*) or sugar (e.g. *Orthomyxoviridae*) motifs used for virus entry, emphasizing that while EVs may facilitate entry in hard-to-infect cells, they can also serve as decoys that reduce infection (Fig. 1-2.4).

Upon binding to target cells, EVs can deliver their cargo to endolysosomal compartments, or fuse with cellular membranes to release their content directly into the cytosol. These two entry routes enable infection by different forms of enclosed virus material. For example, EV–membrane fusion is essential for entry of nonencapsidated viral genomes, whereas entire, mature virions can still make use of their own strategies for membrane penetration after delivery to endolysosomes. Direct cytosolic delivery allows viruses to bypass most entry requirements, however, the overall efficiency of this route and contribution to receptor-independent infection is not known, as current evidence suggests that fusion is rare in the absence of viral fusion proteins on the EV surface (Somiya and Kuroda 2021). For many naked viruses, it is currently still unclear whether capsid disassembly and genome release can even occur when endosomal uncoating triggers are not encountered. Hence, EVs are more likely to promote the bypassing of attachment receptors compared to other entry requirements, aiding virus entry until lysosomal enzymes destroy surrounding EV membranes (shown for *Hepeviridae* and *Picornaviridae*). Equally important to the modulation of direct cell entry is the ability of EVs to influence virus migration through extracellular matrix and tissue barriers (Fig. 1-2.5), a process implicated in enhanced brain-entry efficiency in several families (e.g. *Parvoviridae, Picornaviridae*, and *Flaviviridae*). Improved tissue penetration may in part be supported by enzymes present on the EV surface, such as host neuraminidases, paralleling observations from virions that carry receptor-destroying enzymes (Liu et al. 2025a). Cell wall-degrading enzymes have also been identified on EVs, raising the possibility that EVs similarly promote passage across the type of barriers in encountered in non-mammalian hosts (Silva Barreira da et al. 2023).

#### Antiviral or proviral immune modulation

EVs play an important role in the interaction with antiviral defense mechanisms, either in benefit of the virus or of the host. For instance, vertebrate viruses frequently use EVs to escape from circulating antibodies. This can be achieved by “hiding” in the EV lumen (for both naked and enveloped viruses) (shown for *Astroviridae, Arteriviridae, Parvoviridae, Paramyxoviridae, Togaviridae*, and many more) (Fig. 1-3.1), or by releasing EVs carrying viral membrane proteins as antibody decoys (enveloped viruses only) (e.g. *Coronaviridae, Hepadnaviridae*, and *Filoviridae*) (Fig. 1-3.2). The observed antibody protection in certain cases is only partial, a finding possibly explained by the fact that virions may once again become accessible to antibodies within intracellular compartments after EV membrane rupture (e.g. *Hepeviridae* and *Picornaviridae*). Yet, it remains to be assessed whether the necessary levels of co-internalized antibodies are not an artefact of the high dosages used *in vitro*. EVs also affect direct virus-interactions with the innate immune system, as improved uptake by immune cells of EV-associated viruses compared to free virions has been observed in different settings (Fig. 1-3.3) (e.g. *Togaviridae, Picornaviridae, Hepaciviridae*, and *Hepadnaviridae*), a phenomenon linked to the efficient scavenging of PS-lipids enriched on EVs. As a result, EVs can aid the preferential infection and therefore inactivation of immune cells, but can also promote immune cell activation depending on the tropism of the virus.

Beyond virions, EVs carry viral and host miRNAs, mRNAs, proteins, and lipids with immune modulatory functions. In fact, the sorting of viral cargo into EVs likely relies on strategies similar to those used for host molecules, which may involve recruitment via ribonucleoprotein complexes or the binding to tetraspanin-rich lipid rafts (e.g. *Hepaciviridae, Picornaviridae*, and *Orthoherpesviridae*). A common proposed function of host or viral cargo in virus-induced EVs is gene regulation, particularly through miRNA modulation of antiviral pathways (Fig. 1-3.4) (f.e, *Rhabdoviridae, Arteriviridae, Orthomyxoviridae, Flaviviridae, Hepaciviridae, Phenuiviridae, Retroviridae*, and *Papillomaviridae*). EV transfer of siRNAs directed against different viruses (e.g. *Nodaviridae* and *Togaviridae*) was also proposed, though this requires validation by independent research groups. EVs can play a more direct role in antiviral defense by delivering cytosolic or membrane embedded effector proteins as well as ligands for innate and adaptive immune sensors (Fig. 1-3.5) (e.g. *Orthoherpesviridae, Pseudototiviridae, Retroviridae, Kolmioviridae, Arteriviridae*, and *Orthomyxoviridae*). Although the focus is often placed on the impact of EVs carrying antiviral components on recipient cells, the impact of EV release on the EV-producing cell may be equally relevant. In fact, removing excess host defense factors from infected cells (Fig. 1-3.4), such as mRNAs, reduces their ability to inhibit ongoing virus replication (e.g. *Orthoherpesviridae* and *Paramyxoviridae)*. Finally, it is important to consider the role of uninfected cells, whether nonpermissive, abortively infected or not yet exposed to the virus (illustrated in the context of *Iridoviridae* and *Hepadnaviridae*), because these cells can release EVs enriched in interferon-stimulated genes or other antiviral proteins, the expression of which is often suppressed in infected cells. Findings from *Phycoviridae* infection demonstrate the reverse is also true, and infection can likewise trigger proviral EV production in uninfected neighboring cells, underscoring the importance of reciprocal EV communication during infection.

Currently, a struggle in the field is that different studies often report conflicting proviral and antiviral roles for virus-induced EVs, even within the context of a single or closely related virus species (e.g. *Orthoherpesviridae* and *Phenuiviridae*). In this light, it should be considered that infected cells produce multiple EV types at different infection stages, each carrying distinct cargos (e.g. *Polyomaviridae, Papillomaviridae, Picornaviridae, Asfarviridae, Retroviridae*, etc). Hence, variations in EV isolation and experiment design can lead to the detection of functional differences. Contributing to this effect is the observation that certain host factors upregulated upon infection or inflammation restrict the release of EVs from infected cells (for example tetherin in the case of *Filoviridae* and *Picornaviridae*), which may affect the detected EV populations between cell models, as well as between *in vivo* and *in vitro* settings. “Fine-tuning” mechanisms likely exist to regulate the formation of EVs with opposing functions, however, these remain to be identified.

#### Other (pro-viral) reprogramming of EV recipient cells

While immune modulation tends to claim the spotlight, virus-induced EVs target diverse cellular pathways. One that is of high importance to long-term pathology is malignant cell transformation, as viruses are estimated to cause over 8% of cancer cases (Xiao et al. 2025). Via EVs, the oncogenes that viruses express to promote their own persistence are similarly able to prime uncontrolled growth and survival in uninfected neighboring cells (Fig. 1-4.1, e.g. *Papillomaviridae* and *Orthoherpesviridae*). A related pathway of interest targeted by EVs is the regulation of cell death, particularly apoptosis, which may be blocked to enhance virus production or induced to promote virus release or eliminate antiviral cells (Fig. 1-4.2; e.g. *Phenuiviridae, Polydnaviridae, Papillomaviridae, Flaviviridae, Retroviridae, Pneumoviridae*, and *Filoviridae*). In a more subtle act of trickery, virus-induced EVs promote cell-to-cell transmission by increasing the proximity between infected and uninfected target cells through the modulation of cell migration and cell-cell adhesion (Fig. 1-4.3) (e.g. *Retroviridae* and *Polydnaviridae*). Collectively, studies suggest that EVs promote target-cell susceptibility not only by restricting the efficacy of antiviral pathways (Fig. 1-4.4), but also via mechanisms such as the codelivery of essential host factors for replication (*Potyviridae, Flaviviridae*, and *Orthoherpesviridae*), modulation of endocytic entry pathways (*Adenoviridae* and *Picornaviridae*), or regulation of entry receptor expression (*Paramyxoviridae*). Omics analyses on virus-induced EVs support the identification of such conserved target pathways, although the presence of cargo in EVs does not guarantee a functional response in recipient cells. In addition, functional molecules may be recruited after EV release, as EVs may interact with soluble proteins encountered in the environment, such as viral toxins (implicated for *Flaviviridae*). This in turn can affect potency, as for example demonstrated by the increased bioactivity of retroviral Nef protein when bound to EVs. EV-driven activation of coagulation or complement cascades, as observed in *Orthoherpesviridae* infection, suggests that EVs may similarly promote the recruitment of secreted host factors to target cells. However, further exploration of these phenomena in other contexts is needed to confirm parallels across multiple virus families.

#### Infection-induced alterations in EV-mediated cross-kingdom communication

The exchange of EVs is not limited to individuals of the same species and can even elicit functional responses across species boundaries. As a result, virus-induced EVs modulate not only neighboring cells but also other recipient organisms in a wide variety of settings. In the context of infection, one area in which this is very relevant is the transfer of vector-borne viruses, which alternate in their life cycle between arthropod, plant, and/or vertebrate hosts. EVs were shown to facilitate virus transmission between these different organisms, (Fig. 1-5.1) (e.g. *Flaviviridae* and *Sedoreoviridae*), and initial studies suggest similar exchange of antiviral motifs (*Phenuiviridae*). Although the precise role of EVs in the transmission of vector-borne diseases remains to be fully elucidated, it is tempting to speculate that receptor-independent infection by EVs may aid both vector-borne and zoonotic viruses overcome initial boundaries in the process of adapting to a new host. Also the uptake of EVs by organisms that do not play a direct role in the viral life cycle can be of clinical importance, as evidenced by studies on the impact of virus infection on the exchange of signals between mammals and their microbiota or colonizing microbial pathogens (Fig. 1-5.2) (*Pneumoviridae, Pseudototiviridae, Picornaviridae*, and *Bacteriophage* Pf4). Virus replication in either mammalian or microbial hosts can affect the mutual exchange of EVs, leading to altered immune activation in mammalian carriers or altered growth of colonizing microbial communities. Moreover, such intraspecies crosstalk for *Caliciviridae, Retroviridae*, and *Orthoherpesviridae*, was highlighted to influence virus transmission efficiency. Cross-kingdom EV exchange has also been observed among plants and fungi, indicating that similar findings may arise in even more diverse virus contexts. Different explanations have been proposed for the cross-kingdom effects of virus-induced EVs, including the transfer of EV-bound nutrients, the activation of nucleic acid sensors, or the transfer of regulatory RNA molecules with cross-species target sequences (*Pseudototiviridae, Pneumoviridae*, and *Phenuiviridae*). In addition, EV-mediated dampening of host defenses can indirectly create the possibility for subsequent bacterial or viral pathogens to thrive (shown for *Orthoherpesviridae*). The more diverse the ecosystem, the more complex the interplay between virus and EVs becomes, as viruses do not just alter the EV production in the species in which they replicate (*Caliciviridae*), and may even become part of the EV cargo of non-host species *(Pneumoviridae* and *Picornaviridae*). Fully understanding such interactions will require more studies across biological kingdoms.

#### A word of caution

Establishing patterns of recurring EV release and function (highlighted in Figs 1–3) is important to guide and inspire future studies on virus-host interactions. Especially those EV functions identified across widely varying virus species (Figs 2 and 3) or host kingdoms (Fig. 1) are likely to possess a high degree of biological conservation. Yet, the true scope of this conservation is likely obscured by the fact that findings among infections with different viruses are influenced by hypothesis-driven testing, technical limitations in separating EVs from virions, the choice of EV type studied, and even the selected definition of EVs vs. viruses or VLP. Field-specific preferences in EV isolation protocols or studied subsets also contribute to biases observed across different types of viruses (Figs 2 and 3). For example, concerns about co-isolation of cell debris often lead to the exclusion of time points of virus-induced cell death and/or large EVs, as is a common trend in the study of (nonenveloped) RNA viruses, whereas large EVs have often proven functional and are the primary focus for many studies on DNA viruses. In turn, the EV subset chosen can skew detected functions, as for instance larger EVs more easily provide the dimensions needed for inclusion of entire virions, making them common carriers of infectious viral cargo, while smaller EVs more frequently contain cargo molecules with antiviral properties (seen in Figs 2 and 3). When it comes to the extrapolation of findings, it is important to acknowledge that many uncertainties exist in the translation between experimental and physiological settings, as for example EV dosages tested *in vitro* may not reflect their quantity *in vivo*. The importance of using models that mimic physiological conditions as close as possible has already been shown in the context of *Retroviridae, Orthoherpesviridae*, and *Picornaviridae*, among others, as studies in these areas have shown that physiological cell–cell interactions, the proteome of the selected cell models, and the composition of biofluids in which viruses reside can all affect the predicted functional roles of viral EVs. Nevertheless, an increasing number of *in vivo* studies are being performed that underscore the ability of EVs to impact pathology (*Phycodnaviridae, Nodaviridae, Pseudototiviridae, Sedoreoviridae, Adenoviridae, Parvoviridae, Flaviviridae, Retroviridae, Coronaviridae, Picornaviridae, Arteriviridae*, etc).

## A taxonomy-based overview of EV–virus interactions across the viral kingdom

### RNA viruses

#### Nonenveloped RNA viruses

##### Double-stranded RNA viruses (dsRNA)

###### Giardiaviridae

Giardia lamblia virus (GLV) (species *Giardiavirus ichi*) is the only member of the *Giardiaviridae* (formerly part of the *Totiviridae*), and infects over one-third of all isolates from the highly prevalent gastrointestinal parasite *Giardia duodenalis*. GLV encodes merely two proteins, a capsid and a capsid-RNA-dependent RNA polymerase fusion protein. In the cytosol, these proteins form subviral particles to protect the genome from dsRNA sensors during replication and mRNA synthesis (Marucci et al. 2021). Mature virions (36 nm) are released nonlytically, which is at least in part mediated by EVs based on the observation that nSMase inhibitor GW4869 “traps” virus in the cell, raising intracellular but decreasing extracellular virus loads (Li et al. 2025a). Consistently, EV isolates were shown to carry enclosed viral RNA and upon incubation led to persistent infection (Li et al. 2025a). Giardia-EVs can trigger changes in gene expression in intestinal cell models, however, it remains to be investigated how GLV affects this cross-kingdom response (Yang et al. 2024). Similar totiviruses affecting invertebrates and plants were recently moved to new families, suggesting a possible broader applicability of findings using GLV as model.

###### Pseudototiviridae

*Pseudototiviridae* are small (±5 kb, 30–40 nm) monosegmented viruses that like *Giardiaviridae* used to belong to the reclassified *Totiviridae*, a fact reflected in a similar genome organization. Current recognized members exclusively infect fungi and protozoa. During transmission these viruses typically remain intracellular, spreading only during cell fusion or division, as their virions are sensitive to degradation and lack the machinery for host cell penetration (Hillman and Cohen 2021). However, the observed packaging of viral RNA and capsids within EVs suggests that *Pseudototiviridae* may acquire an EV-envelope to overcome these two barriers to horizontal transmission (Atayde et al. 2019, Ong et al. 2022, Rada et al. 2022). Translation of pseudototiviral genomes in protozoal hosts was confirmed after exposure to membrane-enclosed virions of *Leishmania* RNA virus 1 (LRV1) (species *Leishmaniavirus ichi*), whereas naked virions were taken up less efficiently and were rapidly cleared from cells (Atayde et al. 2019). Similar EV-mediated virus transfer has been observed for *Trichomonas vaginalis* viruses (TVV1-3) (species *Trichomonasvirus vagiprimus, Trichomonasvirus vagisecundus*, and *Trichomonasvirus vagitertious*) (Ong et al. 2022, Rada et al. 2022). Yet, in both settings the infection was unable to persist indefinitely, as is believed to be the case with conventional intracellular pseudototiviridae transmission, suggesting that not all criteria for productive infection are fully met upon EV-mediated virus spread.

As endosymbionts, *Pseudototiviridae* presence is typically neutral to the fungi or protozoa they infect, or even advantageous, as they can provide a competitive growth advantage to their hosts. Mammals carrying *Pseudototiviridae*-infected parasites, on the other hand, show altered immune activation and therefore increased disease severity (Lafleur and Olivier 2022). Virus-driven changes in EV release are proposed to contribute to this effect through EV-mediated cross-kingdom communication. In Leishmaniasis, for example, mice exposed to parasites together with LRV1 virus-induced EVs, develop more severe disease than those receiving EVs from uninfected parasites (Atayde et al. 2019). EVs also participate in virus-driven *Trichomonas*–host interactions, although studies report conflicting pro- or anti-inflammatory effects (Govender et al. 2020, Rada et al. 2022). One mechanism underlying virus-driven pathology is Toll-like receptor 3 (TLR3) activation, which can be triggered by viral dsRNA, the delivery of which is likely facilitated by EVs containing LRV1 or TVV virions (Fichorova et al. 2012, De Carvalho et al. 2019, Govender et al. 2020). Likewise, virus infection also changes the cellular protein and RNA composition of parasite-derived EVs, including the enrichment of tRNA fragments, which may serve as additional TLR ligands and regulators of the mammalian immune response (Atayde et al. 2019, Govender et al. 2020, Rada et al. 2022).

###### Sedoreoviridae

*Sedoreoviridae* are dsRNA viruses consisting of 10–12 genome segments enclosed in 1–3 icosahedral layers of capsid proteins (60–100 nm). They infect a staggering variety of hosts, including mammals, birds, arthropods, algae, and plants. Within this family, rotaviruses are important agents of severe gastroenteritis. Infection with Rhesus rotavirus A (RRV) (species Rotavirus alphagastroenteritidis) increases EV production, primarily of EVs pelleting at low centrifugation speeds (10 000 × *g*, or “10K”), often presumed to represent large EVs (lEVs) (Barreto et al. 2010, Iša et al. 2020). RRV and other mammalian rotavirus A (RVA) strains of the same species have been identified either inside or attached to such vesicles in cell culture and stool samples (Barreto et al. 2010, Iša et al. 2020). Although VP4 (the outer capsid spike protein of RVA) proteolytic processing is typically considered a requirement for efficient RVA infection, virions within EVs are also found in unprocessed form in certain cell models, leading to speculation that this proteolytic activation step is less important if entry occurs through EVs. Notably, virus-containing EV-associated virions appeared in the intestine shortly after fecal–oral transmission, and were more pathogenic than equivalent amounts of naked virions upon oral administration (Santiana et al. 2018). Combined with the fact that EVs also reduce antibody-mediated neutralization, these findings prompt further investigation of the role of EVs in RVA pathogenesis (Iša et al. 2020).

Another important virus of the *Sedoreoviridae* family is Bluetongue virus (BTV) (species Orbivirus caerulinguae), which infects mostly sheep and goats through Culicoides midge species. BTV is a nonenveloped virus but can still bud from the plasma membrane through a nonstructural membrane protein interacting with the capsid and the ESCRT-associated protein TSG101 (Labadie et al. 2020). This budding is especially important in insect hosts where BTV does not cause lysis, blurring the boundaries between naked, enveloped, and EV-bound viruses. In parallel, BTV appears to trigger the release of infectious EVs from intracellular membrane sources, biogenesis of which involves trafficking through MVBs and de-acidified lysosomes, and relies on autophagic or ER-degradation pathways for the recruitment of viral cargo (Labadie and Roy 2020, Wu et al. 2025). The resulting lEVs and sEVs carry one or multiple luminal virions, and differences in infectious potential and infection dynamics are observed when enriching for the different forms of infectious particles. African horse sickness virus (species Orbivirus alphaequi), a virus known to infect both mammals and insects, was also seen budding at the plasma membrane, however only during mammalian cell infection (Venter et al. 2014).

Plant Rice dwarf virus (RDV) (species Phytoreovirus alphaoryzae), which uses various species of leafhoppers for its transmission, similarly modulates EV release. It was demonstrated that cultured leafhopper cells release larger EVs during RDV-infection (114–307 nm) than those secreted by uninfected cells (58–138 nm) and these EVs contain large amounts of viral particles. For successful insect-borne horizontal transmission, RDV hijacks an EV release pathway involving MVB to cross the plasmalemma and enter the salivary cavities of its arthropod vector. The exploitation of this EV biogenesis pathway allows transfer of EV-enclosed virus from the insect into the phloem of rice plants to establish initial viral infection (Wei et al. 2008, 2009, Chen et al. 2021). Notably, EV packaging of RDV virions in leafhopper cells appears to involve interactions between the viral capsid and Ras-related protein 5 (Rab5), a vesicular transport protein localized to endosomes. Together with the upregulation of Rab27a and CD63, this indicates active manipulation of MVB-mediated EV biogenesis (Chen et al. 2021). It is of note that other phytoreoviruses, like Rice gall dwarf virus (RGDV) (species Phytoreovirus betaoryzae) and wound tumor virus (species Phytoreovirus vulnustumoris) have also been identified in MVB of insect cells, suggesting potential EV-mediated release (Shikata and Maramorosch 1965, Mao et al. 2017). In addition, RGDV was shown to induce autophagy to promote transmission, a finding that warrants examination of autophagy-mediated loading of virus into EVs (Chen et al. 2017).

###### Spinareoviridae

Viruses of the family *Spinareoviridae* closely resemble *Sedoreoviridae* members, but their typically dual-layered capsid (50–85 nm) contains spikes. Mammalian orthoreovirus (MRV) (species *Orthoreovirus mammalis*) is a virus that infects humans but rarely causes disease. It has been demonstrated that MRV increases the production of EVs, with primarily lEVs (400–600 nm) containing one or more viral particles either inside or on their surface, resulting in a partial resistance to antibody neutralization (Smith et al. 2024). Using barcoded viruses, it was confirmed that these lEVs enhance the coordinated infection of cells by multiple virions, generating mixed genetic variants within single plaques (Smith et al. 2024). Southern rice black-streaked dwarf virus (species *Fijivirus boryzae*), a plant pathogen, also accumulates virions in large autophagy-related vesicles but whether virions are released together with these membranes remains to be formally demonstrated (Zhang et al. 2023). In contrast, for avian reovirus (ARV) (species *Orthoreovirus avis*), a “fusogenic” virus that induces syncytia formation and affects the poultry industry, the packaging of viral cargo into sEVs (pelleted at 100 K) has been reported (Duncan et al. 1996, Wang et al. 2023). These sEVs were shown to contain viral proteins, including those capable of triggering cell-fusion, but no complete virions. Still, virions co-isolated with these EVs are substantially more infectious than ARV alone (Wang et al. 2023). Since low-speed EV pellets were excluded from analysis, it is not known whether similar production of larger, infectious EV subsets by ARV occurs. However, a role for EVs, and even secretory autophagy, in ARV transmission is not unlikely, given observations that knockdown of tetraspanins CD81 and CD63 inhibit virus release, along with a positive correlation between virus release and overall autophagy levels (Hsu et al. 2025). Finally, an unassigned reovirus from the parasitoid wasp *Hyposoter exiguae* (*H. exiguae* reovirus, HeRV) appears to exit cells in membrane-enclosed form via plasma membrane budding, resembling the unconventional release also observed for the insect-borne *Sedoreoviridae* BTV, though the nature of the resulting particles requires further study (Stoltz and Makkay 2000).

##### Positive single-stranded RNA viruses (ssRNA+)

###### Astroviridae

*Astroviridae* are ± 30 nm monopartite RNA viruses that commonly cause gastroenteritis in mammals and birds, as well as occasional neurological disease. Moreover, the detection of cross-species recombination events highlights the risk for astroviruses as potential zoonotic pathogens (Roach and Langlois 2021). Human astrovirus (HAstV) (species *Mamastrovirus hominis*) preferentially increases the release of EV pelleting at 20 K over other denser EV subsets (Baez-Navarro et al. 2022). These vesicles confer partial resistance to neutralizing antibodies, indicating that HAstV virions are released in EV-enclosed form before cell lysis. Although astroviruses typically require capsid maturation by intra- and extracellular proteases for transmission (Banos-Lara and Méndez 2010), EVs allow both enclosed and surface-associated virions to enter cells without prior trypsin activation (Baez-Navarro et al. 2022). In fact, depending on the recipient cell and protease availability, EVs were found to either enhance or limit HAstV spread, highlighting a context-dependent role of EVs in astrovirus infection (Baez-Navarro et al. 2022).

###### Caliciviridae

*Caliciviridae* are 27–40 nm viruses targeting a wide range of vertebrates. Among these, feline calicivirus (FCV) (species *Vesivirus felis*) was found to stimulate EV production (Mizenko et al. 2022). Although also co-isolating with EVs, true FCV EV-envelopment is yet to be confirmed, as was demonstrated for human norovirus (NV) and murine norovirus (MNV-1) (species *Norovirus norwalkense*) in phosphatidylserine (PS)-enriched EV subsets from cell culture and stool samples (Santiana et al. 2018). The nonlytic release of murine norovirus could be repressed by nSMase inhibitor GW4869, supporting an endosomal source of virus-carrying EVs during norovirus infection and possibly that of other caliciviruses. EV-enclosed MNV-1 showed increased UV resistance, suggesting a functional role for EVs in environmental stability, potentially mediated by an increased cooperation between virions that acquire harmful mutations (Zhang et al. 2021). Furthermore, EVs appear to mediate a complex interaction between caliciviruses and bacterial bystander species. Physical interaction between MNV-1, NV, and intestinal bacteria induces stress responses that boost production of small bacterial extracellular vesicles (BEVs) (Mosby et al. 2022). Modulation of BEV release was confirmed in the intestine of mice infected with MNV-1, and is proposed to activate inflammatory pathways controlling MNV-1 replication (Bhar et al. 2022, Mosby et al. 2022). Specific bacterial effector proteins as well as BEV-associated nucleic acids have been implicated in the priming of MNV-1 resistance, contributing to observations that BEV from different bacterial sources appear to rely on (partially) distinct cellular sensors to restrict MNV-1 replication, including DNA sensor STING and endosomal TLRs (Silva da et al. 2024, Zhao et al. 2026).

###### Dicistroviridae

*Dicistroviridae* are specialized arthropod viruses belonging to the larger order of picornavirales, and affect for example bees, ants, aphids, but also shrimps and crabs. Among these, cricket paralysis virus (CrPV) (species *Cripavirus grylli*) shows nonlytic virus release from *Drosophila* cells with the release of both EV and non-EV associated virus (±30 nm) detected before loss of membrane integrity (Kerr et al. 2018). Released EVs purified via density gradient-based purification were found to contain mature, infectious virions, but lacked nonstructural viral proteins. Moreover, quantitative changes in host-derived EV protein content were observed (Kerr et al. 2018). These findings suggest that already at an early stage of infection, CrPV is capable of triggering changes in EV composition or release, including the selective incorporation of virions, the mechanism behind which remains subject to investigation.

###### Hepeviridae

*Hepeviridae* are monosegmented RNA viruses infecting mammals, birds, and fish. The most studied member is hepatitis E virus (HEV) (species *Paslahepevirus balayani*), typically causing acute hepatitis, although cases of chronic infections have been reported (Pallerla et al. 2020). HEV particles (±30 nm) identified in feces are nonenveloped (nHEV), whereas virions circulating in blood and cell culture supernatant are typically surrounded by lipid membranes (Takahashi et al. 2008). As a result, HEV is considered quasi-enveloped (eHEV), since its default form appears enveloped, but this envelope is not required to establish infection. eHEV particles exhibit similarities to EVs since various EV markers decorate their surface, alongside a Golgi protein (Nagashima et al. 2014, 2017). HEV localization within MVBs suggests that viral packaging likely occurs through budding in the endosomal compartments, a finding supported by the ability of nSMase and Rab27 inhibitors to block HEV release (Nagashima et al. 2014, Glitscher and Hildt 2021). Typically, only one virion is present in EVs carrying HEV. Additionally, both viral ORF2 and ORF3 proteins are present in eHEV, while nHEV particles carry only ORF2 (Takahashi et al. 2008). In fact, palmitoylated ORF3 was found essential for EV-mediated packaging of HEV virions through its interaction with annexin A2 and ESCRT-associated proteins (Liu et al. 2025b). Additionally, infected cells were found to release a substantial fraction of ORF3 protein (up to 40%) within EVs that lacked infectious viral material (Liu et al. 2025b). The EV packaging of HEV enables the evasion of patient-derived antibodies but simultaneously delays viral entry due to a relatively inefficient cell adhesion and the need for lysosomal degradation of EV membranes to enable cytosolic penetration (Yin et al. 2016, Chapuy-Regaud et al. 2017). Proteins required for the degradation of the eHEV envelope include Niemann-Pick disease type C1-protein and lysosomal acid lipase, both involved in lipid metabolism (Yin et al. 2016). Although the precise entry requirements for eHEV are still being elucidated, there appears to be an important role for EV membrane lipids and corresponding cellular receptors, including PS-receptor TIM-1 (Chapuy-Regaud et al. 2017, Corneillie et al. 2023).

###### Nodaviridae

*Nodaviridae* (±30 nm) are segmented, positive-sense RNA viruses that primarily infect insects (Alphanodaviruses) and fish (Betanodaviruses), though their flexible host range has enabled studies in suckling mice as a model. In mice, an attenuated nodamura virus (NoV) strain (species *Alphanodavirus nodamuraense*) has been used to study the possible contribution of EVs from infected cells to (systemic) antiviral immunity, specifically via the cell-to-cell spread of antiviral small interfering RNA molecules (siRNA). EVs enriched from the plasma of NoV-inoculated mice were found to carry virus-directed siRNA molecules capable of transmitting antiviral activity restricted to viruses with complementary RNA sequences, however, this finding awaits independent validation (Zhang et al. 2022a). If confirmed, this opens up a role for EVs in a unique, systemic RNA-mediated immune response, a process that is perhaps even more relevant in organisms that lack other forms of adaptive immunity, e.g. insects and plants. EV involvement in the biological cycle of another nodavirus has also been proposed, namely that of the red-spotted grouper nervous necrosis virus (RGNVV) (species *Betanodavirus epinepheli*), a virus causing significant losses in the aquatic industry. Pull-downs with the host factor Alix (implicated in EV and in particular exosome biogenesis) in cell extracts revealed co-precipitation of the RGNVV viral capsid, hinting at the existence of a possible sorting mechanism of RGNVV virions into (s)EVs (Yu et al. 2024). Although an increase in sEV secretion during infection was found, a corresponding presence of virions within the lumen of isolated EVs awaits further technical validation at this time.

###### Picornaviridae

The *Picornaviridae* family includes over 147 species infecting various animals. Viruses in at least six genera package viral material in EVs, indicating widespread EV-virus interactions. EVs primarily incorporate mature picornavirus particles (±30 nm), ranging from 1 to almost 20 virions per EV (Feng et al. 2013, Robinson et al. 2014, Chen et al. 2015, Yang et al. 2020) and may contain low levels of nonstructural viral components (Zhang et al. 2019, Yang et al. 2020, Van Der Grein et al. 2022). Besides encapsulated virions, EVs can transfer nonencapsidated picornavirus genomes and viral particles bound to their surface, suggesting a diverse role for EVs in picornavirus spread (Fu et al. 2017, Costafreda et al. 2020, Rudy et al. 2022, Fu and Xiong 2023).

Hepatitis A virus (HAV) (species *Hepatovirus ahepa*) was one of the first viruses discovered to use EVs for virus release *in vitro* and *in vivo*. In serum, EV-enclosed, or “quasi-enveloped” HAV virions (eHAV) are the predominant form of virus particles, whereas naked virions are more prevalent in fecal samples, presumably due to a bile-mediated loss of surrounding membranes (Feng et al. 2013, Hirai-Yuki et al. 2016, Karamichali et al. 2022). An endolysosomal origin for eHAV has been identified based on protein composition and involvement of ESCRT-associated proteins in eHAV formation (Feng et al. 2013, McKnight et al. 2017). In fact, two peptide motifs (YXXL) in the viral capsid, one of which is uniquely retained in eHAV but cleaved off for naked HAV, were shown to directly interact with the ESCRT-protein Alix to promote eHAV packaging (Feng et al. 2013, González-López et al. 2018, Jiang et al. 2020a, Shirasaki et al. 2022). These HAV motifs resemble “late domains” implicated in the budding of multiple enveloped virus species, causing eHAV to blur the boundaries between naked and enveloped viruses (Welker et al. 2021).

Contrary to HAV, studies on coxsackievirus B3 (CVB3) (species *Enterovirus alphacoxsackie*), enteroviruses poliovirus (PV) (species *Enterovirus coxsackiepol*), and cardiovirus encephalomyocarditis virus (EMCV) (species *Cardiovirus rueckerti*) have shown that EV packaging heavily relies on secretory autophagy. EV-enclosed virions appear prior to cell lysis and coincide with enhanced incorporation of the lipidated form of the autophagy marker microtubule-associated protein 1 light chain 3 (LC3-II) (Robinson et al. 2014, Chen et al. 2015, Grein van der et al. 2019, Giansanti et al. 2020, Van Der Grein et al. 2022, Zhang et al. 2022a). Inhibiting autophagosome formation, or mitophagy in the case of CVB3, reduces nonlytic and/or EV-mediated virus spread (Zhang et al. 2011, Bird et al. 2014, Chen et al. 2015, 2025, Sin et al. 2017, Zhang et al. 2022a), although data interpretation is often complicated since autophagy also supports viral replication. The other way around, the pharmacological activation of the secretory arm of the autophagy pathway in EMCV infection has also been shown to increase EV-mediated virus release (Van Der Grein et al. 2022). The host factor transcription factor EB acts as a specific regulator of this pathway during CVB3 infection, however, it remains to be investigated whether different picornaviruses share the same regulatory strategies (Giansanti et al. 2020). In the search for positive regulators of EV-mediated release, it is important to consider that virus packaging in EVs may not be limited to a single pathway. For example, EMCV still releases EV-enclosed virions even when secretory autophagy is impaired (Van Der Grein et al. 2022). In line with this premise, multiple distinct EV subsets carrying viruses have been identified in EMCV and PV-infected samples, highlighting the heterogeneity of EV-associated picornaviruses (Yang et al. 2020, Van Der Grein et al. 2022). In a striking example, CVB5 virions were even detected by EM imaging in vesicle-like structures produced by amoebae, adding further to the possible complexity of encountered EV-enclosed virions (Atanasova et al. 2018). A second important consideration is that different virus isolates can show variation in the extent to which they rely on EV-mediated spread, based on observations among different HAV strains and the demonstrated ability of CVB3 to evolve toward more or less use of EVs during transmission (Feng et al. 2013, Bou and Sanjuán 2020).

Quantitative and qualitative changes in EV production often accompany the EV packaging of picornavirions. In addition to the species mentioned above, this has also been reported for viruses such as duck hepatitis A virus (DHAV) (species *Avihepatovirus ahepati*), echovirus 16 (E16) (species *Enterovirus betacoxsackie*), enterovirus D68 (EV-D68) (species *Enterovirus deconjuncti*), and rhinovirus (RV) (species *Enterovirus alpharhino)* (Chen et al. 2015, Huang HI et al. 2020, Netanyah et al. 2020, Jassey et al. 2023, Xu et al. 2023). While HAV capsid proteins directly engage EV-biogenesis machinery, other viruses may employ nonstructural viral proteins and/or host cell signaling pathways to influence EV release. For instance, EMCV L and CVB3 2A proteins enhance virus-carrying EV subsets by affecting cellular stress pathways, particularly those involving host kinases PKR and P38 MAPK (Defourny et al. 2024). Overall, it is important to consider that EV release during infection varies between EV subsets and infection stage, and can even be negatively regulated (van der Grein et al. 2019, Xu et al. 2023). For instance, foot-and-mouth disease virus (FMDV) (species *Aphthovirus vesiculae*) infection reduces EV release through downregulation of Rab27a (Xu et al. 2020a). Likewise, host factors can counteract EV release, as tetherin inhibits EV release during enterovirus 71 (EV71) (species *Enterovirus alphacoxsackie*) infection (Fu et al. 2017), and CVB3 infection can trigger antimicrobial peptide release that disrupts EV membranes (Yang et al. 2023).

Picornavirus-derived EVs play conflicting roles in pathogenicity. In *in vivo* infection models EV-enclosed EV71, as well as CVB3 virions in the presence of EVs, show higher viral loads, morbidity, and mortality, whereas EV-FMDV or eHAV delay mortality and/or virus shedding (Das et al. 2017, Zhang et al. 2019, Gu et al. 2020, Fu et al. 2023, Tian et al. 2025). Yet, it is a common finding that EV enclosure provides a competitive advantage against neutralizing antibodies, as also seen with Seneca Valley virus (species *Senecavirus valles*) (Feng et al. 2013, Mao et al. 2016, Grein van der et al. 2019, Zhang et al. 2019, Netanyah et al. 2020, Xu et al. 2020b, Fu et al. 2023, Xu et al. 2023). This functional diversity may reflect differential packaging of pro- and antiviral factors, with demonstrated examples including proviral miRNAs inhibiting IFN-β induction and apoptosis, or antiviral regulators targeting retinoic acid-inducible gene 1 (RIG-I) and endocytic pathways (Fu et al. 2017, Germano et al. 2019, Wu et al. 2019a, Xu et al. 2020b). Another explanation is the MOI difference between EV isolates, as for PV and CVB3 it was demonstrated that by delivering multiple virions to one cell, EVs, and especially lEVs, reduce the number of target cells (Chen et al. 2015, Bou and Sanjuán 2020, Yang et al. 2020). Yet at the same time, such lEVs enhance coinfection and early viral protein production, as shown for PV and EV71 (Chen et al. 2015, Yang et al. 2020, Tian et al. 2025). Alongside direct effects on the host, study of RV infection shows that virus-induced EVs can even affect the growth of colonizing microbes, specifically the biofilm formation of *Pseudomonas aeruginosa*, a finding likely linked to EV cargo changes triggered by antiviral pathways (Hendricks et al. 2021).

Finally, EVs also change picornavirus uptake dynamics and tissue targeting. At a systemic level, EV-enclosed EV71 was shown to cross brain barriers more efficiently than its naked counterpart (Gu et al. 2020, 2023, Tian et al. 2025). On a cellular level, EVs decrease the efficiency of CVB3 attachment to host cells (Bou and Sanjuán 2020). Simultaneously, PS-lipids on EVs may improve virus uptake by immune cells, promoting infection for EV-enclosed PV but triggering immune activation in the case of eHAV (Chen et al. 2015, Feng et al. 2015, Das et al. 2017). Transfer of PS + EVs to antiviral immune cells such as plasmacytoid dendritic cells (DC) is strongly enhanced by direct contact with infected cells, as was shown for eHAV, indicating that *in vitro* models may fail to fully recapitulate uptake dynamics *in vivo* (Feng et al. 2015). Importantly, EVs also affect the uptake of naked virions. CVB3 and EV-D68 were shown to bind to attachment factors on EV surfaces (Rudy et al. 2022, Fu and Xiong 2023, Fu et al. 2023). For CVB3, these EVs could facilitate infection of receptor-negative cells, enhancing replication in tissues like the brain and immune cells (Fu and Xiong 2023, Fu et al. 2023). The ability of EVs to mediate receptor-independent transfer of enclosed picornaviruses is still a matter of debate, as infection typically seems to require EV membrane degradation in (endo)lysosomes and subsequent receptor-dependent entry into the cytosol, similar to that discussed for HEV (indicated for HAV and PV). This can delay infection and allows cointernalized antibodies or receptor depletion to inhibit virus entry (Feng et al. 2013, Chen et al. 2015, Rivera-Serrano et al. 2019). EVs containing nonencapsulated viral RNA bypass typical entry requirements, a phenomenon implicated in receptor-independent transfer of EV71 via sEVs and possibly also lEVs, the former of which carried the viral RNA in complex with the RNA-binding protein Argonaute 2 (Fu et al. 2017, Tian et al. 2025). However, EV fusion efficiency is typically extremely limited in the absence of viral fusion proteins (Somiya and Kuroda 2021). Moreover, such RNA-containing EVs may trigger innate immune sensors if RNA is released in endosomes instead, as demonstrated for Theiler’s murine encephalomyelitis virus (species *Cardiovirus theileri*) (Luong and Olson 2021).

###### Potyviridae

Potyviruses are filamentous plant viruses (up to 900 nm long and 20 nm wide), with 8–11 kb RNA genomes. In 2019, Movahed et al. (2019) demonstrated that turnip mosaic virus (TuMV) (species *Potyvirus rapae*) infection reorganizes intracellular membranes to generate vesicles within structures resembling MVBs. These MVBs displayed enhanced association with the plasma membrane during infection, resulting in amplified numbers of vesicles in the extracellular space and translocation over the cell wall. It was suggested that these MVBs and resulting EVs were decorated with viral proteins, viral RNA, and various host proteins required for viral replication, although it remains unclear whether these components are inside the vesicles or simply adhering to their surface. A recent publication suggested an altered miRNA profile in EVs from potato virus Y (PVY) (species *Potyvirus yituberosi*) infected tomato plants compared with healthy controls, the targets of which suggest a possible role for EVs in the innate defense response during infection. However, also here the absence of controls for the localization of these miRNAs inside the EVs, as opposed to their association with non-vesicular particles, compromises data interpretation (Wang et al. 2026). Potyvirus components were also identified in plant-derived nanovesicles (PDNV) of tomato tissue, i.e. a heterogeneous mix of intra and extracellular vesicles extracted by or created during tissue homogenization. A combination of MS-based proteomics and cryoTEM analysis identified different tomato viruses, including PVY, within isolated PDNVs. Based on these findings, the authors hypothesized that the identified viral proteins could also be secreted extracellularly via PDNVs (Mammadova et al. 2021).

#### Enveloped RNA viruses

##### Positive single-stranded RNA viruses (ssRNA+)

###### Arteriviridae

*Arteriviridae* are veterinary pathogens, including the Porcine reproductive and respiratory syndrome virus (PRRSV) (species *Betaarterivirus europensis*), which poses a great economic burden in the swine industry. Arteriviruses are enveloped spherical viruses (50–74 nm), containing a genome of ± 12–16 kb. During PRRSV infection, CD63 + sEVs containing full-length viral RNA and a limited set of viral proteins form EV–virus hybrid structures (Montaner-Tarbes et al. 2016, Wang et al. 2018, Cheng et al. 2023). Unlike free virions, these EVs could propagate infection to cells lacking the typical entry receptor in both the absence or presence of neutralizing antibodies, and were sensitive to inhibition by GW4869 (Wang et al. 2018). During acute infection, changes in host-derived EV cargo, including metabolites and microRNAs (miRNAs), have also been observed, highlighting virus-driven modulation of EV-producing and possibly EV-recipient cells (Montaner-Tarbes et al. 2016, Cheng et al. 2023). In addition to facilitating immune escape, EVs can play a role in immune priming in later stages of PRRSV infection, as evidenced by the display of immunogenic viral antigen on EVs from nonviremic or convalescent animals. These EVs could enhance cellular immunity more effectively than synthetic viral peptides, suggesting their potential as alternative vaccine candidates (Montaner-Tarbes et al. 2018). Moreover, in the case of equine arteritis virus (species *Alphaarterivirus equid*), a specific role for EVs in viral persistence was indicated, as seminal EVs from long-term carriers contained decreased levels of eca-mir-128, which is linked to increased expression of the chemokine CXCL16, a positive predictor of persistent infection (Carossino et al. 2018).

###### Coronaviridae

Coronaviruses (CoVs) are zoonotic viruses that primarily cause respiratory and gastrointestinal disease in both human and animals. The most well-known of these is severe acute respiratory distress syndrome (SARS)-CoV-2 (species *Betacoronavirus pandemicum*), which is responsible for the COVID-19 pandemic. CoVs have a helical nucleocapsid surrounded by a spherical lipid bilayer (80–160 nm in size) and have four structural proteins, the N (nucleocapsid protein), M (Membrane), E (envelope), and S (Spike transmembrane glycoprotein). Their monopartite RNA genome can reach 33.5 kb, the largest among positive single-stranded RNA viruses (ssRNA+ viruses) (Fehr and Perlman 2015). Increasing evidence suggests that CoVs hijack the endocytic pathway involved in EVs biogenesis, altering EV content. Infection commonly leads to accumulation of virus material in structures resembling late endosomes or lysosomes, as well as induction of lysosomal exocytosis and lysosomal deacidification, all of which may influence EV secretion (Ghosh et al. 2020). Xia et al. (2023) showed that in cell cultures, SARS-CoV-2 envelope protein induced the release of large EVs (1–10 μm) containing one or more infectious viral particles, capable of escaping neutralizing antibodies and establishing infection independent of typical entry receptors. Other studies using cell lines and/or patient-derived EVs (plasma and sputum) have detected smaller EVs (30–125 nm) that similarly contained N and S proteins, as well as viral genome (Barberis et al. 2021, Pesce et al. 2022, Sun et al. 2023), with differences between studies likely reflecting variation in employed cell types, viral strains, and EV isolation methods. The presence of S proteins on EVs was shown to efficiently activate immune responses *in vitro* by enhancing CD4^+^ T-cell activation (Pesce et al. 2022) but at the same time Troyer et al. (2021) revealed that S-carrying EVs from cell lines act as decoys for neutralizing antibodies, indicating a complex interplay between the and EVs over the course of infection.

During SARS-CoV-2 infections, patients often develop a hyperactive immune response syndrome (termed “cytokine storm”) corresponding to the release of cytokines, chemokines, and other inflammatory proteins (Fajgenbaum and June 2020). Various EV proteome studies repeatedly showed high levels of immune, inflammatory, coagulation, and complement-related proteins, implicating EVs in COVID-19 pathogenesis (Barberis et al. 2021, Sun et al. 2023). Furthermore, EVs have been shown to carry viral entry factors, such as Angiotensin-converting enzyme 2 (ACE2) and the spike-priming transmembrane protease serine 2 (TMPRSS2), especially during infection. This allows them to act either as antiviral decoys, or conversely to enhance infection by transferring these proteins to recipient cells, including those that naturally lack them, such as alveolar macrophages and endothelial cells (El-Shennawy et al. 2022, Tey et al. 2022, Rea-Moreno et al. 2026). SARS-CoV-2 may further exploit EVs and other endogenous lipid carriers such as high-density lipoproteins, to cross the blood–brain barrier (Lam et al. 2022). Lipidomics analysis revealed that serum lipid signatures linked to COVID-19 disease severity closely resembles those found in EV membranes, including enhanced levels of sphingomyelin (SM) and ganglioside GM3, indicating GM3-enriched EVs might contribute to the disease (Song et al. 2020). Interestingly, altered EV composition and abundance persists long after acute infection in a condition called post-covid or “long” covid, suggesting a role for EVs in sustaining this condition (Dalle Carbonare et al. 2025, Oliveira de et al. 2025).

Finally, other CoVs also manipulate EV pathways. Cells infected with canine coronavirus and feline coronavirus (species *Alphacoronavirus suis*) were shown to produce moderately more EVs compared to control cells. The levels of ACE2, flotillin-1, CD44, and other host proteins linked to inflammation and cell stress were altered in EVs after infection revealing a virus-dependent cargo change, but further investigations are necessary to elucidate the mechanism (Pandit et al. 2023, Wijerathne et al. 2025). Expanding on the type of cargo analysed, the formation of spike-decorated EVs containing an altered composition of host RNAs was demonstrated for porcine hemagglutinating encephalomyelitis virus (PHEV) (species *Betacoronavirus 1*), a neurotropic coronavirus infecting pigs (Li et al. 2022). Only selective strains of PHEV are sensitive to inhibition with GW4869, suggesting that the interaction with EV pathways is variable and not intrinsically conserved (Zaib et al. 2025). Similar to PHEV, viral proteins and RNA have been identified inside sEVs for porcine epidemic diarrhea virus (PEDV) (species *Alphacoronavirus porci*). Importantly, for PEDV these EVs mediated resistance to neutralizing antibodies and were infectious both in susceptible and nonsusceptible cell lines, suggesting a possible new transmission mode for this virus (Ding et al. 2023). While these findings increase the diversity of host species in which EV–coronavirus interactions are being uncovered, significant gaps remain regarding the full spectrum of species implicated in the zoonotic transmission of coronaviruses and the potential role of EVs in this process.

###### Hepaciviridae

*Hepaciviridae* target a range of vertebrate species but are best studied in the context of the human pathogen hepatitis C virus (HCV) (species *Orthohepacivirus hominis*). Until recently, members of this family were classified within *Flaviviridae*, and therefore share substantial genomic and biological similarities with classical flaviviruses. For HCV, the formation of infectious sEVs have been described both *in vivo* and *in vitro* (Masciopinto et al. 2004, Tamai et al. 2012, Ramakrishnaiah et al. 2013, Liu et al. 2014). These sEVs acquire infectivity through incorporation of nonenveloped viral genomes or whole viral particles, as was corroborated by (T)EM, the use of envelope-deficient replicon viruses, and the resistance to neutralizing antibodies (Masciopinto et al. 2004, Tamai et al. 2012, Ramakrishnaiah et al. 2013, Liu et al. 2014). In addition, enrichment of HCV coreceptor CD81 on EV membranes may promote virion binding to the EV surface, on top of its demonstrated ability to promote the sorting of HCV envelope proteins into EVs (Masciopinto et al. 2004).

Encapsulation of HCV RNA within the EV lumen has important functional consequences, most notably the ability to initiate receptor-independent infection, as was shown for EVs carrying HCV RNA complexed with the RNA-binding protein Argonaute 2 (Bukong et al. 2014). However, uptake by otherwise nonpermissive cells also poses disadvantages since EVs containing HCV RNA and other possible pathogen-associated molecular patterns activate innate immune responses in dendritic cells, unlike cell-free virions (Dreux et al. 2012). Among the immunogenic cargo in EVs are HCV–dsRNA replication complexes, the incorporation of which is promoted by binding to membrane proteins such as the ER-resident protein Reticulon-3 (Li et al. 2020a). At the same time, release of such complexes via EVs is proposed to restrict their delivery to RNA sensors in endolysosomal compartments of the producing cell (Grünvogel et al. 2018). Beyond full-length viral genomes, EVs are also proposed to transport DVGs, which are frequently generated during chronic *Hepaciviridae* infections and can modulate viral replication efficiency (Karamichali et al. 2018). At the moment, the relative contribution of EVs carrying different types of viral RNA cargo to the establishment of long-term, persistent infection remains unclear, although the uptake of various forms of HCV is likely to contribute to the lower RNA-to-PFU ratio observed for EVs compared to virions (Liu et al. 2014). Adding further complexity, HCV proteins associated with EVs display posttranslational modifications that differ from those found in virions, opening up the possibly for particle-specific functions for these proteins (Liu et al. 2014). Finally, also the cellular EV cargo is of importance, with miR-122 worthy of particular notice. Found in EVs in complex with HCV–RNA, this miRNA is believed to enhance viral translation efficiency and inhibit natural killer cells (Bukong et al. 2014, Karamichali et al. 2022).

Several ESCRT-related (TSG101 and CHMP4B) and other proteins previously associated with EV production, such as Annexin A2, have been implicated in the biogenesis of EVs containing HCV viral motifs. In addition, it was demonstrated that viral RNA transfer via EVs can be suppressed by nSMase inhibitors without interfering with viral RNA replication (Dreux et al. 2012). In contrast, syntenin-based EV biogenesis pathways have specifically been implicated in the release of noninfectious EVs containing HCV envelope protein E2, which acts as an antibody decoy to promote infection (Deng et al. 2019). HCV virions have been described in MVB structures, despite a typical ER-mediated virus exit, although it can be challenging to validate whether virions detected here are undergoing EV-dependent or independent release (Tamai et al. 2012). Also, upon treatment with cholesterol transport inhibitor U18666A a retention of virions in CD63+ compartments has been described, supporting the possible use of such compartments as a secretory route (Elgner et al. 2016).

###### Flaviviridae

*Flaviviridae* are a family of monopartite, enveloped viruses (9–13 kb; 40–60 nm particles), that cycle between arthropods and humans. EVs from both arthropods and mammalian cells infected with dengue virus (DENV) (species *Orthoflavivirus denguei*), zika virus (ZIKV) (species *Orthoflavivirus zikaense*), and Japanese encephalitis virus (JEV) (species *Orthoflavivirus japonicum*) have been proposed to contain viral proteins, RNAs, viral genomes and/or even complete virus particles, based on the detection of membrane-enclosed viral envelope proteins (E), genomic RNA, and/or VLP within infectious EV isolates (Li et al. 2020b, Reyes-Ruiz et al. 2020, York et al. 2021, Calderón-Peláez et al. 2025, Xiong et al. 2025). These EVs have been found to promote antibody-resistant infection across species, and in some cases increase cellular uptake and infectivity, based on the ratio between RNA and plaque-forming units (pfu) (Li et al. 2020b, Reyes-Ruiz et al. 2020, Xiong et al. 2025). There are reports even suggesting that EVs help viral RNA cross barriers into hard-to-reach tissues, like the placenta and the brain, on top of facilitating viral transfer within these tissues, as shown for JEV and ZIKV, with EVs from JEV-infected cells showing a higher brain-entering capacity than those from uninfected cells (Calderón-Peláez et al. 2025, Xiong et al. 2025).

Beyond enhancing flavivirus transmission, EVs carrying noninfectious viral cargo shape infection in various ways. During DENV/ZIKV infection, EVs containing viral membrane proteins act as antibody decoys while simultaneously limiting antibody-dependent enhancement of infection (Zhao et al. 2023). In insects, EVs may support immune evasion, by packaging proviral, subgenomic RNA molecules (sfRNA) (Yeh et al. 2023). These sfRNAs are formed through partial degradation of the viral genome by host cell machinery, and aid in the escape from RNA interference pathways, among other functions. In addition to the uptake of viral cargo during EV biogenesis, studies on viral toxin NS1 demonstrate that EVs can even sequester soluble virus proteins encountered in the environment (Safadi et al. 2023). Although the impact of this sequestration remains unclear, contrasting effects on IFN pathways have been reported for cells overexpressing tick-borne encephalitis virus (species *Orthoflavivirus encephalitidis*) NS1 versus those treated with NS1-carrying EVs (Kuzmenko et al. 2025).

When interpreting the role of EVs in different flavivirus infection models, it should be noted that infection may trigger the release of EVs with distinct characteristics in cells of the two different species in question (mosquito vs. human) (Reyes-Ruiz et al. 2020). Analysis of EV properties indicates that along with the packaging of viral cargo, the size of produced EVs changes during infection and that in some cases, like tick-borne Langat virus (species *Orthoflavivirus langatense*) infection, the quantity of EVs is also affected, suggesting differences in the manipulation of EV biogenesis among family members (Zhou et al. 2018, Reyes-Ruiz et al. 2020). Contributing to species-specific differences is the uptake of a repertoire of cellular factors in EVs, whose roles in most cases remain unidentified. The abundance of over 30 miRNAs was found to be changed in EVs from DENV patients with more severe disease manifestation, highlighting their possible role as biomarker for disease progression. Supporting a role for EV-bound miRNAs in DENV pathogenesis, predicted targets include numerous cytokine induction or signaling pathways (Agudelo et al. 2025). Another process frequently targeted is cell death. MiRNAs in DENV3-EVs may promote apoptosis of dendritic cells (Martins et al. 2018), while those in JEV-EVs promote activation of caspases in bystander neurons (Mukherjee et al. 2019). In addition to miRNAs, virus-induced EVs carry mRNAs potentially capable of producing relevant proteins in target cells. For instance, EVs from West Nile virus (WNV) (species *Orthoflavivirus nilense*) infected cells contain the mRNA coding for DDX58, a protein essential for restricting viral infection (Slonchak et al. 2019), while DENV-EVs include mRNAs for cytokines CXCR4 and IL-8, which are linked with Dengue shock syndrome (Martins et al. 2018). Finally, important proteins are carried in EVs, including IFN-inducible transmembrane protein 1 and 3 (IFITM1 and IFITM3), known for their antiviral effects, in DENV-EVs (Zhu X et al. 2015).

Lipid metabolism acts as an important regulator for *Flaviviridae* virus replication and budding, as well as EV release. For ZIKV, infection promotes the expression and activity of nSMase-2/SMPD3 while LGVT suppresses arthropod sphingomyelinase D (SMase D), highlighting two alternative strategies used by *Flaviviridae* to modify the levels of SM and its product ceramide, a known regulator of EV biogenesis (Zhou et al. 2019b, Regmi et al. 2020). Lipids, and most notably PS, in the viral envelope also play an important role in viral attachment to host cells, and as a result host-derived EVs enriched in PS can interfere with the entry of viruses like ZIKV, DENV, and WNV, by occupying PS-recognizing cell receptors, potentially explaining low infectivity in body fluids like semen and breast milk despite ample virus presence (Groß et al. 2024)

Another pathway involved in EV-mediated viral release is autophagy. Modulation of autophagy, from autophagosome formation to cargo degradation, has been well documented for the *Flaviviridae* family (Ke 2018). DENV and ZIKV infections were shown to drive the release of EVs carrying the autophagosome marker LC3 in a trafficking pathway regulated by the host Lyn kinase, and depleting this EV subset reduced DENV transmission (Teo et al. 2021, Cloherty et al. 2024). More in-depth characterization revealed that viral material is likely packaged in EVs within amphisomes, hybrid organelles between autophagosomes and MVBs (Teo et al. 2021). However, a full characterization of the role of secretory autophagy in EV formation and/or cargo loading is needed for each *Flaviviridae* species, as their interactions with the autophagy pathway may vary (Ke 2018, Teo et al. 2021).

###### Pestiviridae

The recently defined family *Pestiviridae* includes viruses that infect mammals such as swine and ruminants. These viruses can cause a range of symptoms, including hemorrhagic disease, abortion, and severe mucosal disease. These viruses strongly resemble *Flaviviridae* and are enveloped with spherical geometries (∼50 nm) and a linear genome of around 12 kb. Within this family, classical swine fever virus (species *Pestivirus suis*) was found to produce infectious sEVs that contain a mix of viral RNA, structural, and nonstructural viral proteins, and are capable of initiating an antibody-resistant infection (Bao et al. 2024). Supporting a role for EVs in virus transmission, viral proteins E0 and E1 were shown to interact with Rab27a to increase EV production for enhanced viral spread (Li et al. 2025b). Furthermore, a mechanistic role for autophagy was established since inhibiting autophagy significantly reduced EV-mediated virus export (Wang et al. 2022). A first characterization hints to differences in the transcriptional response of cells exposed to virus samples before or after enriching for EVs, warranting further assessment of the role of EVs in immune activation (Bao et al. 2024).

###### Togaviridae

Chikungunya virus (CHIKV) (species *Alphavirus chikugunya*), Sindbis virus (SINV) (species *Alphavirus sindbis*), and Mayaro virus (species *Alphavirus mayaro*) are among the more than 30 species of alphaviruses in the *Togaviridae* family. These small enveloped viruses (50–90 nm) are typically transmitted between mosquitoes and vertebrates (land-based and aquatic), although other arthropods and arthropod-exclusive alphaviruses have been noted (Chen et al. 2018). Antibody-resistant transfer of alphaviruses is frequently linked to viral transmembrane proteins that form cell protrusions, known as tunneling nanotubes, facilitating receptor-independent virus transfer (Yin et al. 2023). A complementary role of EVs in this process is still under investigation, and is supported by evidence that sEV-rich preparations from CHIKV-infected cells can mediate antibody-resistant transmission (Le et al. 2022). A special focus is warranted for ApoBD EV subsets during *Togaviridae* infection, as SINV capsids have been found in vesicles from virus-induced apoptotic cells, and also for CHIKV a decrease in virus spread was observed upon the blocking of apoptotic blebbing (Rosen et al. 1995, Krejbich-Trotot et al. 2011). In fact, large ApoBDs (>0.22 μm) were shown to transmit CHIKV to macrophages more efficiently compared to smaller extracellular virus fractions (Krejbich-Trotot et al. 2011). Paradoxically, host-derived EVs rich in PS-lipids, which often include ApoBDs, simultaneously act as natural decoys for *Togaviridae*, and specifically CHIKV, by competing with virions for receptor binding (Groß et al. 2024). Overall, reports of virions in structures resembling EVs go back decades, as is the case for Mayaro virus (Mezencio et al. 1989), but further work is needed to fully understand the interaction of *Togaviridae* with different EV subsets. This not only includes the role of EVs in virus transmission but also that in antiviral immunity, since SINV-derived siRNAs detected in EV-enriched plasma suggest a possible function for EVs in systemic antiviral RNA interference, as was demonstrated in more detail for nodamuravirus (see *Nodaviridae*) (Zhang et al. 2022b).

##### Negative single-stranded RNA viruses (ssRNA-)

###### Filoviridae

The *Filoviridae* family includes ebola virus (EBOV) (species *Orthoebolavirus zairense*), one of the most virulent pathogens known to humans. Filoviruses are characterized by their unique long filamentous structure (∼1 μm in length) and contain a ssRNA genome of about 19 kb. *Filoviridae* viral proteins bind directly to and alter the expression levels of ESCRT proteins (including Alix and TSG101), thereby potentially manipulating EV biogenesis pathways and contributing to the detection of EBOV proteins (mainly VP40) within EVs *in vitro* and *in vivo* (Pleet et al. 2018, 2019). VP40-positive EVs were shown to induce apoptosis in T-cells, potentially accelerating disease progression (Pleet et al. 2018). Additionally, EVs carrying viral VP40/NP may contribute to immunopathology by activating macrophages, as was demonstrated for EV samples containing a mix of EVs and VLP, an effect enhanced when EBOV viral GP glycoprotein was present (Vucetic et al. 2023). In contrast, EV samples containing only viral GP were found to inhibit the release of cytokines, including C-C Motif Chemokine Ligand 2/5 (CCL2/5) and tumor necrosis factor-alpha (TNF-α) (Nehls et al. 2019) .In addition to modulating cytokine induction, EVs from VP40-expressing cells also act as direct cytokine carriers, such as TGF-β, IL-15, MCP-1, and IFN-γ, all linked to the cytokine storm in severe EBOV infection (Pleet et al. 2018). These findings suggest a multifactorial role for EVs in the modulation of the host immune system depending on the combination of cargo molecules.

Finally, the release of EVs, specifically those carrying viral envelope GP protein, is believed to deplete EBOV-neutralizing antibodies (Nehls et al. 2019). *In vivo*, EV release is increased throughout EBOV infection, with GP + EVs in serum appearing at later time points, coinciding with a trend for increased EV size (Pleet et al. 2018, Vucetic et al. 2023). Consistently, GP expression was shown to stimulate the release of CD81 + lEVs *in vitro* (Nehls et al. 2019). At the same time, there are indications that the host tries to prevent the release of such particles. Specifically, the protein tetherin was shown to keep GP + EVs tethered to the cell surface, the strain-dependent efficiency of which correlated with pathogenicity (Nehls et al. 2019). Direct interactions between GP and tetherin facilitate the efficiency of this process compared that for other EV subsets, and enable the host to use tetherin to simultaneously inhibit the release of EBOV virions, pathological EVs, and EV–virus hybrid particles as a strategy to slow the infection.

###### Orthomyxoviridae

*Orthomyxoviridae* is a family of segmented ssRNA-viruses, including the well-known influenza A/B viruses (IAV/IBV) (species *Alpha- and Betainfluenzavirus*) (80–120 nm). Orthomyxoviruses infect mammals, birds, salmon, and various arthropods, causing significant mortality during seasonal, pandemic, and zoonotic outbreaks (McGowan et al. 2019). These viruses replicate in the nucleus and are released via budding, typically killing the host cell (Neumann et al. 2004). Electron microscopy studies indicate that during budding, host proteins like EV markers tetraspanin CD9 and CD81 may be incorporated into the IAV envelope (Shaw et al. 2008). Conversely, viral proteins and RNAs are frequently identified in EVs, complicating the discrimination between these two particles (Atkin-Smith et al. 2020, Bedford et al. 2020, Zabrodskaya et al. 2022, Ao et al. 2024). In spite of this challenge, EVs have been suggested to contribute to IAV spread by transferring viral ribonucleoprotein complexes, surface-bound, or intraluminal virions, or even by serving as a platform for ongoing virus budding after virus-induced (apoptotic) cell death (Atkin-Smith et al. 2020, Ao et al. 2024). At the same time, EVs also have antiviral functions. EVs released during infection were found to trigger immune responses, including neutrophil infiltration and the generation of antigen-specific T cells (Atkin-Smith et al. 2020, Bedford et al. 2020). These responses could occur in the absence of productive infection mediated by EVs, implicating other EV functions or cargo in this process. In fact, EVs can transfer viral HA antigens for processing and MHC-II presentation, among others, and presentation of HA on EVs also enhances transfer and presentation of other antigens in the same particle (Testa et al. 2010).

Upon exposure to IAV, increased production of small and/or large EVs is seen *in vitro* and *in vivo* (Cypryk et al. 2017, Maemura et al. 2018, Bedford et al. 2020). Alterations in EV release were shown to peak during viral replication, but compositional changes *in vivo* persisted well after virus clearance (Maemura et al. 2018, Bedford et al. 2020). These alterations include changed RNA profiles, with upregulated antiviral factors like miR-483–3p and proinflammatory Y5 RNA-derived small RNAs, but also antiviral and proinflammatory cytokines and autophagy-related proteins, although the role for autophagy in IAV-EV cargo loading remains to be investigated (Cypryk et al. 2017, Bedford et al. 2020, Jiang et al. 2020b, Zabrodskaya et al. 2022, Kwasnik et al. 2023). Conversely, IAV infection can lead to proviral changes in EV cargo, including the increased uptake of miRNAs that inhibit antiviral proteins and the presence of specific small nuclear RNAs (snoRNAs) linked to the infection (Jiang et al. 2020b, Zabrodskaya et al. 2022, Rozek et al. 2025). In addition to EVs from infected cells, EVs from bystander cells may also inhibit infection, depending on the combination of cell type and virus strain (Bedford et al. 2020, Jiang et al. 2020b, Schneider et al. 2020). This inhibitory activity primarily stems from the presence on influenza attachment factors such as α-2,3 and α-2,6-linked sialic acids on EVs, enabling them to act as decoys to reduce virus uptake (Bedford et al. 2020, Jiang et al. 2020b). Alternatively, it was proposed that EV-enriched pellets from alveolar macrophages are able to inhibit the pH-dependent fusion step of IAV (Schneider et al. 2020). Together, these findings suggest that EVs influence multiple stages of infection, from virus entry to resolution.

###### Paramyxoviridae

*Paramyxoviridae* infect a range of vertebrates, including mammals, reptiles, fish, and birds, and are responsible for highly contagious diseases such as measles, mumps, and various respiratory infections in humans. These enveloped virions (150–500 nm) contain a negative-sense ssRNA molecule of about 15 kb, and are commonly transmitted through respiratory droplets. One notable member, the Newcastle disease virus (NDV) (also known as Avian paramyxovirus 1) (species *Orthoavulavirus javaense*), causes substantial losses in the poultry industry. EVs released from NDV-infected cells incorporate viral proteins but typically cannot transmit infection directly. Instead, these EVs enhance virus replication and cytopathogenicity in cells subsequently exposed to free virions (Xu et al. 2019, Zhou et al. 2019a). In contrast, EVs from caprine parainfluenza virus type 3 (CPIV3) (species *Respirovirus caprae*), and peste des petits ruminants virus (PPRV) (species *Morbillivirus caprinae*) can mediate direct virus transmission, which is notably more efficient than conventional virus spread and helps evade antibody neutralization (Mao et al. 2020, Chen et al. 2022, Wan et al. 2022).

Both viral and host EV cargo contribute to the ability of *paramyxoviridae* EVs to enhance infection. PPRV-EVs, for example, transfer viral proteins that modulate PPRV entry receptor expression and suppress cytokine release (Chen et al. 2022). Similarly, the priming activity of NDV-EVs can be reproduced by the EV-associated viral NP protein (Xu et al. 2019). In addition, EVs from NDV and CPIV3 infections are enriched in proviral miRNAs that inhibit autophagic degradation of virions as well as IFN-β production (Zhou et al. 2019a, Mao et al. 2020). Beyond the delivery of proviral cargo, EVs can also serve as an excretion pathway of antiviral molecules. For instance, NDV-EV mediates secretion of NLRX1 mRNA, reducing protein levels and thereby diminishing the antiviral state of infected cells (Xu et al. 2021).

Across paramyxoviruses, infection consistently increases EV release (Zhou et al. 2019a, Mao et al. 2020, Xu et al. 2021, Chen et al. 2022, Wan et al. 2022). Although the underlying mechanisms remain largely unknown, EV induction is often sensitive to GW4869 treatment (Xu et al. 2021, Chen et al. 2022, Wan et al. 2022). In addition, a positive correlation was observed between TSG101 levels and PPRV virus packaging into EVs, the expression of which is regulated by multiple virus proteins during infection (Wan et al. 2022). Finally, the ability of NDV to induce apoptosis prompts further investigations into the release and role of lEVs, and specifically ApoBDs EV subsets in infection (Sun et al. 2014).

###### Phenuiviridae

*Phenuiviridae* contains members infecting insects, birds, humans, plants, and fungi. Most are enveloped (80–120 nm), with segmented ssRNA-genomes (two to eight segments), except for the nonenveloped (filamentous) Tenuivirus genus. Despite their diverse hosts, all members replicate in the cytoplasm, bud in Golgi cisternae and exit via the conventional secretory pathway. Rift Valley fever virus (RVFV) (species *Phlebovirus riftense*) is a mosquito-borne virus causing severe hemorrhagic illness in ruminants and sometimes humans (Ahsan et al. 2016). All three genomic segments (L, M, and S) as well as viral proteins (N, NSs, and Gn/Gc) were identified in EVs derived from acute or persistently infected cells (Ahsan et al. 2016, Alem et al. 2021). Yet, these EVs are not infectious, and instead modulate immunity either by RIG-1-mediated induction of IFN-β, or by inducing apoptosis of immune cells. EVs from dabie bandavirus (also known as severe fever with thrombocytopenia syndrome virus) (species *Bandavirus dabieense*), a tick-borne virus causing high fever, diarrhea, and leukocytopenia in humans, also carry NSs protein, but unlike RVFV, can contain intact infectious virions capable of antibody-resistant infection spread, suggesting a novel EV-mediated transmission route (Silvas et al. 2016). Another interesting case is rice stripe virus (RSV) (species *Tenuivirus oryzaclavatae*). RSV is transmitted in rice plants through the salivary glands of small brown planthoppers during their feeding. Lu et al. (2022), found that EVs from the saliva of virulent insects are enlarged and carry infectious virions. The host protein exportin 6 was shown to facilitate incorporation of RSV particles into EVs, suggesting a selective route for virus release (Lu et al. 2022). At the same time, rice-derived EVs were recently shown to enter midgut epithelial cells of the small brown planthopper and deliver specific miRNAs to interfere with RSV infection. These miRNAs include osa-mir167a, which targets the RSV RdRp, and osa-mir159a, which modulates phospholipase C expression, thereby inhibiting apoptosis (Wang et al. 2025a). This represents the first documented cross-kingdom, EV-mediated interaction between plants and insects to restrict viral infection.

###### Pneumoviridae

*Pneumoviridae* are large negative-sense RNA viruses (13.2–15.3 kb) infecting mammals and birds. Most notorious among them is respiratory syncytial virus (RSV) (species *Orthopneumovirus hominis*), which causes acute respiratory tract infections in the elderly, children, and immunocompromised individuals. RSV infection triggers increased release of sEVs, which harbor viral RNA and proteins but cannot initiate infection (Chahar et al. 2018, Hendricks et al. 2021, Corsello et al. 2022). Instead, they carry antiviral cytokines and chemokines, as well as various mRNAs, noncoding RNAs, miRNAs, and piwi-interacting RNAs that support antiviral activity by regulating transcription, translation, and apoptosis in the receiving cell (Chahar et al. 2018, Corsello et al. 2022). However, RSV-EVs can also exacerbate health issues by promoting the growth of secondary pathogens like *Pseudomonas aeruginosa*, a phenomenon linked to increased levels of iron-binding protein transferrin on RSV-EVs, facilitating bacterial uptake of this essential nutrient (Hendricks et al. 2021). Another example of complex, interkingdom interactions is the interaction between RSV and amoebae, which can internalize environmentally encountered RSV particles and package these virions into released EVs (Dey et al. 2021). During this process infectivity is retained, and consequently amoebae may promote environmental persistence of RSV particles based on observations of increased stability of EV-associated viruses made for other viruses.

###### Rhabdoviridae

*Rhabdoviridae* comprises bullet-shaped viruses (up to 430 nm in length and 100 nm in width) with a negative-sense RNA genome capable of infecting plants and animals, including arthropods. Rabies virus (RABV) (species *Lyssavirus rabies*) stimulates sEV production in Vero cells. Although typical strategies for inhibiting EV release reduce extracellular viral loads, their potential effects on virus replication preclude definitive conclusions regarding a possible proviral role for EVs in this context (Wu et al. 2019a). In RABV-infected MRC-5 cells, however, production of sEVs containing high levels of mir-43–5p was observed, which inhibits viral replication by targeting suppressor of cytokine signaling 3 (SOCS3) (Wang et al. 2019a). Another interesting case recently reported is that of viral hemorrhagic septicemia virus (VHSV) (species *Novirhabdovirus piscine*), a highly pathogenic virus infecting a Pacific Ocean fish (olive flounder). It was shown that plasma EVs from infected fish presented different miRNA populations compared to healthy counterparts, linked to enhanced immunomodulatory activities upon EV injection (Nikapitiya et al. 2025). Furthermore, these VHSV-EVs were found to have an altered proteome profile, containing proteins important for wound-healing (Nikapitiya et al. 2025). Finally, the species Spodoptera frugiperda rhabdovirus (species *Betapaprhavirus frugiperda*), a virus identified as a contaminant in a significant number of sf insect cell lines, has been studied in relation to EVs. It has been shown that the viral protein G of this virus is present in isolated EVs, though its role needs further investigation (Van Es et al. 2024). Ιn EV research, the viral G proteins of rhabdoviruses such as vesicular stomatitis virus (species *Vesiculovirus indiana*) or chandipura virus (species *Vesiculovirus chandipura*) are frequently used to load specific proteins and nucleic acids inside EVs, a technique known as “exosomes pseudotyping” (Meyer et al. 2017, Zhang et al. 2024b), suggesting that its natural incorporation into EVs during infection may be biologically significant. Future research will likely reveal more roles of EVs in rhabdovirus biology, such as for the maize mosaic virus (species *Alphanucleorhabdovirus maydis*), which has been observed within MVBs (Ammar and Nault 1985).

##### Circular RNA viruses (circRNA)

###### Kolmioviridae

Viruses of the *Kolmioviridae* family have a negative-sense circular RNA genome enclosed in an envelope typically produced by a helper virus, resulting in 36–43 nm particles. Members affect mammals, birds, reptiles, and insects. Hepatitis delta virus (HDV), classified under the species *Deltavirus italiense*, is the most well-known virus of this family. Together with its helper virus Hepatitis B virus (HBV), they are responsible for severe inflammatory liver disease, which can rapidly progress to cirrhosis and hepatocellular carcinoma (Jung et al. 2020). sEVs isolated from HDV-infected cells induce a dose-dependent cytokine production, contributing to the strong inflammatory response unique to HDV–HBV coinfection (Jung et al. 2020). During coinfection, HDV proteins trigger an increase in EV release not observed in the sole presence of HBV, and are also detected in sEVs (Khabir et al. 2024). Via their interaction with the viral genome, these proteins are also proposed to contribute to observations of HDV RNA EV-packaging (Jung et al. 2020, Khabir et al. 2024). It is of note that HDV-like viruses have been described that appear capable of replicating without a (known) helper virus, raising the possibility that EVs are used as the sole endogenous transmission route for this viral family (Paraskevopoulou et al. 2020).

##### RNA viruses with reverse transcribed DNA intermediates (RNA-RT)

###### Retroviridae

*Retroviridae* carry a positive-sense viral RNA genome, which is transcribed into dsDNA and integrated into the host genome. Their envelopes carry a sparse collection of unevenly distributed viral proteins, the formation of which blurs the boundaries between virus and EV biogenesis (Nolte-‘t Hoen et al. 2016). In fact, detailed proteomic analysis has indicated the selective uptake of several EV proteins into virions and *vice versa* during infection (Martin‐Jaular et al. 2021), highlighting the complexity of studying mixtures of these particles. Human immunodeficiency virus 1 (HIV-1) (species *Lentivirus humimdef1*) has been extensively studied, revealing the production of different types of EVs containing viral envelope proteins, nonstructural proteins, and small RNAs (Martin‐Jaular et al. 2021, Lee 2023, Boucher et al. 2025). However, because EVs and virions share components, purity concerns should always be considered in data interpretation. Proviral functions are primarily attributed to EVs containing HIV viral motifs, whereas cellular EV cargo has been implicated in a mix of pro- and antiviral effects (recently reviewed in Lee 2023). Depending on their origins (mammalian or even bacterial), EVs can either inhibit or facilitate uptake of coincubated virions, and affect infection susceptibility through EV-induced signaling (Costantini et al. 2021, Lee 2023). The binding of virions to the surface of EVs, a key step in modulating virus entry, was shown to be facilitated by transcytosis, a process during which virions are internalized into MVB and subsequently exocytosed together with EVs, especially in immature DC (Wiley and Gummuluru 2006). Overall, a key take-away appears to be that EV heterogeneity is critical when considering function in HIV infection. For instance, EVs from infected T cells or DCs differ in their ability to promote HIV infection due to varying protein compositions (Kulkarni and Prasad 2017).

In addition to modulating infection, EVs carrying HIV gene products contribute to different aspects of pathology, including apoptosis of CD4 T cells and chronic immune activation (Lee 2023). The viral protein Nef is a key player in this process and is detected in EVs during HIV-1 and simian immunodeficiency virus (SIV) (species *Lentivirus simimdef*) infection, even in patients receiving effective antiretroviral therapy (DeMarino et al. 2018, McNamara et al. 2018, Vanpouille et al. 2024). Multiple motifs are linked to the efficient packaging of Nef into EVs, including binding to Alix, myristoylation, and binding to chaperones (Buffalo et al. 2019). Nef-EVs are taken up by myeloid cells, altering cholesterol metabolism and increasing lipid rafts, thus regulating HIV-cell fusion and contributing to HIV comorbidities like long-term immune hyperreactivity (Sviridov et al. 2020, Dubrovsky et al. 2022). EV-bound Nef is over 100-fold more bioactive than its soluble form, highlighting the importance of EVs in transferring viral virulence factors (Vanpouille et al. 2024). However, also the role of host-derived EV cargo should not be overlooked, as changes in EV miRNA content were linked to SIV neuropathology via the activation of the RNA sensor TLR-7 in the brain (Yelamanchili et al. 2015).

Also for human T-cell Lymphotropic Virus Type-1 (HTLV-1) (species *Deltaretrovirus priTlym1*), viral RNA and proteins are detected in EVs from infected cells or patients (Pinto et al. 2019, 2021). Although not infectious, these EVs are capable of enhancing infection spread *in vitro* and *in vivo* (Pinto et al. 2019). Specifically, HTLV-1 EVs trigger immune cell clustering via EV-bound CD45 and ICAM (intercellular adhesion molecule), facilitating virus spread through direct cell contact between infected and uninfected cells (Pinto et al. 2019, 2021). HTLV-EV composition varies with pelleting speeds (2, 10, or 100 K), regarding both cellular and viral factors. For instance, autophagy-related markers are specifically found in larger EVs from low-speed pellets (Pinto et al. 2021). Moreover, subtle differences in the functional properties of these EV populations were observed, including the tissues in which they affected infection dynamics. Similar variations in EV cargo and function have also been reported among HIV EV-subsets isolated via differential centrifugation steps, indicating this is a phenomenon researchers ought to be generally aware of (Boucher et al. 2025). The HTLV-1 protein Tax1 was found to directly bind to the syntenin-1, a regulator of exosomal biogenesis, shedding light on a possible mechanism by which HTLV-1 affects at least part of the detected EV subsets (Puttemans et al. 2025).

Finally, reticuloendotheliosis virus (REV) (species *Gammaretrovirus aviretend*), an avian retrovirus transmitted via semen, uses EVs to establish infection in an antibody-resistant manner (Su et al. 2021). REV co-isolated with CD63 + EVs from infected animals transmitted infection more efficiently than purified virus, a finding that coincided with lower induction of various immune regulators upon initial virus exposure (Su et al. 2021). A similar phenomenon is seen during HIV infection, though for HIV, but not REV, infection could be blocked by targeting both EV and viral surface proteins (Wiley and Gummuluru 2006, Kulkarni and Prasad 2017), indicating differences in the molecules facilitating EV entry, or a difference in virus localization to the inside or outside of EVs. Presence of viral genomic RNA in CD63 + seminal EVs was also shown for the subgroup J avian leukosis virus (ALV-J) (species *Alpharetrovirus avileu)*. Interestingly, only EV isolates, but not free virions, could transmit ALV-J to hens and their eggs in the presence of semen, despite insemination with similar numbers of infectious virus units and a comparable infection efficiency *in vitro* (Liao et al. 2022). Hence, further exploration of the importance of EVs in retrovirus inter-individual spread is of particular interest.

### DNA viruses

#### Nonenveloped DNA viruses

##### Double-stranded DNA viruses (dsDNA)

###### Adenoviridae

*Adenoviridae* are typical icosahedral viruses (90 nm) with a capsid marked by twelve long, protruding filaments. Their 25–48 kb genome can accommodate large foreign segments making it a favored carrier in gene editing and vaccine development. Adenoviruses (AdV) infect a range of vertebrate species, from turtles to birds and mammals, and cause prevalent infections in humans. During AdV-induced pneumonia, a distinct miRNA profile was detected in serum-derived EVs (Huang et al. 2019). Similar alterations in EV-enclosed small RNAs, most notably that of miRNAs and snoRNAs, were observed for lung epithelial cells infected with human adenovirus type 4 (HAdV-E4) (species *Mastadenovirus exoticum*), with predicted consequences for the regulation of viral translation, endocytic and apoptotic pathways (Noghero et al. 2025). These RNA changes coincide with reduced incorporation of antiviral-related proteins into EVs. Additionally, small noncoding RNAs of viral origin have been identified in EVs with known proviral functions, a finding also confirmed with oncolytic adenovirus strains (Brachtlova et al. 2024, Noghero et al. 2025). Currently, the precise role of EVs in AdV-induced disease remains unsolved. In both mouse and cell models, knockdown of nSMase2, Rab27, or Alix increased AdV viral loads, which is possibly linked to antiviral EV functions (Li et al. 2013, Takahashi et al. 2017). The proposed mechanisms include EV-mediated disposal of viral DNA, and propagation of type-I IFN responses between nonpermissive and permissive cells. At the same time, EVs were found to enable infection by transferring AdV receptors to nonsusceptible cells, potentially broadening tissue tropism (Gonzalez et al. 2012, Sims et al. 2014).

The functional interplay between AdV and EVs is best characterized for genetically engineered oncolytic adenovirus (OA) vectors. These vectors induce lytic, proinflammatory cell death in target tumor cells, a property that is mimicked by EVs isolated from OA-infected cells (Ran et al. 2016, Saari et al. 2020, Kakiuchi et al. 2021, Feng et al. 2024). Notably, EV-enclosed OA virions maintain efficacy despite prior virus exposure or presence of antisera, and kill tumor cells more effectively than free virions, including receptor-negative and stem-like tumor cells (Ran et al. 2016, Feng et al. 2024). Additionally, their antitumor efficacy is increased by the induction of synergistic modulation of immune cells, which is mediated by the EV particle itself, especially those particles released by infected cells (Feng et al. 2024). In particular, a role for improved B-cell activation was proposed, mediated by the enrichment of relevant miRNAs in OA-carrying EVs (Feng et al. 2024). Even when using non-EV-enclosed OA virions as therapeutic agents, the EVs generated within patients upon virus exposure contribute to systemic treatment efficacy. Local OA virus injections in primary tumors were shown to shrink secondary tumors, a process inhibited by the nSMase inhibitor GW4869 (Kakiuchi et al. 2021). This antitumor effect is linked to the EV-mediated transfer of viral and host cargo to secondary tumor tissues, a site preferentially targeted by these EVs, and is independent of adaptive immune activation (Kakiuchi et al. 2021). Further characterization is needed to confirm the parallels with natural infection settings. Nevertheless, it is reasonable to assume that EVs likewise modulate AdV spread in other contexts. In fact, EVs were recently proposed to contribute to the transmission of infectious HAdV-E4, a natural HAdV pathogen (Noghero et al. 2025). However, the type of infectious material transferred (e.g. EV-associated mature virions vs. genomic DNA in a subviral context) requires further confirmation, as both options have also previously been suggested for OA EV preparations (Ran et al. 2016, Saari et al. 2020).

###### Iridoviridae

*Iridoviridae* is a family of icosahedral dsDNA viruses infecting fish, amphibians, reptiles, insects, and crustaceans. Virions contain an internal lipid membrane but do not require an outer envelope for infection. *Iridoviridae* release both enveloped virions via budding at the plasma membrane and nonenveloped virions (120–350 nm) in the form of paracrystalline arrays during cell lysis (Chinchar et al. 2017). Whether the enveloped forms carry EV markers is still unknown. Iridoviruses encode both anti- and pro-apoptotic factors to finely regulate cell death and virus dissemination (İnce et al. 2018). This results in the production of lEVs (ApoBDs) that carry progeny virus particles and are phagocytosed by neighboring cells, as shown during late red sea bream iridovirus (species *Megalocytivirus pagrus1*) infection (Imajoh et al. 2004). Apart from facilitating virus transfer, EVs can also protect against iridovirus infection. For example, serum-derived EVs from mandarin fish cells reduced infection levels of infectious spleen and kidney necrosis virus (species *Megalocytivirus pagrus 1*) *in vitro* due to the presence of the interferon-inducible protein Mx1 (He et al. 2021). Similarly, EVs from salamander testis protected cells against Chinese giant salamander iridovirus infection (species *Ranavirus rana*) (Gao et al. 2018).

###### Marseilleviridae

*Marseilleviridae* are large viruses (250 nm), containing a circular dsDNA genome up to 400 kb, and are often referred to as “giant” viruses. Virions are commonly detected in water samples, and infect mostly amoebae, though their presence in insects, mussels, and humans suggests that their host range is probably broader (Sahmi-Bounsiar et al. 2021). In the case of marseillevirus marseillevirus (MsV) (species *Marseillevirus massiliense*), it was shown that viral release during cell lysis produces either individual viruses or clusters of particles enclosed in membranes potentially originating from ER. These clusters have not yet been formally classified as an EV subset, although their morphology fits the criterion of an EV and parallels observations in other virus families, where EVs facilitate the release of multiple virions as a single, membrane-enclosed infectious unit. Compared to free virions, these vesicles present MsV with several advantages, namely increased infectivity on a per particle basis and an increased tolerance to high temperatures (Arantes et al. 2016). Additionally, they were found to use different entry routes (Arantes et al. 2016). Khalifeh et al. (2022) investigated megavirus genomes and suggested the existence of virus-encoded eukaryotic vesicle trafficking factors, including SNARE-like proteins and related machinery such as Rab proteins, through which these viruses may affect EV release. Similar factors were recently identified in bacterial pathogens, suggesting that appropriating such host proteins is a conserved and sophisticated strategy among intracellular pathogens to manipulate vesicle trafficking (Khalifeh et al. 2022).

###### Papillomaviridae

Viruses of the *Papillomaviridae* family are icosahedral (55 nm), with a dsDNA genome of 5–8 kb. They infect fish, reptiles, birds, and mammals. Human papillomaviruses (HPV) (species *Alphapapillomavirus* 1, 2, and 3, etc.) are sexually transmitted and are responsible for 5% of all cases of cancer globally (De Martel et al. 2017). Various studies have analysed the connection of EVs with HPV. First, it has been clearly demonstrated that HPV infection increases sEVs production in cell lines and tumors (Honegger et al. 2013, Ludwig et al. 2018, Mata-Rocha et al. 2020, Nguyen et al. 2020, Ranjit et al. 2020). Second, these EVs contain viral RNA, DNA, and proteins, with particular emphasis on viral E6/E7 (Ludwig et al. 2018, Mata-Rocha et al. 2020, Nguyen et al. 2020). Silencing of the coexpressed viral oncogenes E6/E7 strongly modulates the host cargo composition of EVs from HPV + cell lines, highlighting their key role in sEV modulation during (Honegger et al. 2013, 2015). Among the host proteins preferentially loaded in EVs, survivin, a protein involved in apoptosis inhibition, is of particular interest given its significance as a poor prognostic marker in cervical cancer (Honegger et al. 2013). A collection of EV-enclosed miRNAs and mRNAs with a similar role in the inhibition of apoptosis and cervical carcinogenesis was also identified, which altogether likely contribute to the confirmed ability of sEV-enriched patient plasma samples to promote migration and survival of tumor cells (Tong et al. 2020, Yan et al. 2025). In patient settings, the immune signature of EVs also appears important in pathology, since HPV presents with a proinflammatory cargo profile in sEV-enriched plasma, loss of which was correlated with the gain of immune suppressive properties and cancer-status. A notable example of relevant molecules includes IFITM2, an important antiviral protein (Yan et al. 2025). Although focus is often placed on sEVs, it should be considered that HPV-integrated cells are also implicated in the transfer of oncogenic viral and host material to their neighbors via the release of lEVs upon cell death (ApoBDs) (previously reviewed in Guenat et al. 2017).

###### Phycodnaviridae

EVs appear to have a beneficial role in *Phycodnaviridae* infection, which represents a family of large dsDNA viruses (100–220 nm particles with a 120–440 kb genome) infecting marine or freshwater eukaryotic algae. Cells infected with Emiliania huxleyi virus (species *Coccolithovirus huxleyi*) produce increased numbers of EVs with a unique lipid and a distinct small RNA cargo composition, which enhances viral propagation within algal blooms. This in turn leads to the release of large amounts of organic matter and nutrients, reshaping carbon and nutrients flow through the marine microbial food web and affecting all present microorganisms (Schatz et al. 2017, 2021). Infected cells were found to stimulate similar EV production in bystander cells, expanding their effect (Schatz et al. 2017). Mechanistically, EV-associated small RNAs are thought to have regulatory functions and prime the cell for subsequent viral infection, accelerating infection dynamics (Schatz et al. 2017, 2021). Increased EV abundance further enhances the environmental stability of released virions, thereby promoting virus spread; however, the underlying protective mechanism remains unclear (Schatz et al. 2017).

###### Polyomaviridae

Polyomaviruses are small (40–45 nm) viruses that infect mammals, birds, and fish. These viruses replicate and assemble within the nucleus, establish long-term persistent infections and are released either lytically or nonlytically (Moens et al. 2017). Various viruses of this family have been associated with the production of both small and large EVs containing one or more virus particles, such as JC polyomavirus (JCPyV) (species *Betapolyomavirus secuhominis*) and BK polyomavirus (BKPyV) (species *Betapolyomavirus hominis*) (Morris-Love et al. 2019, 2022, Handala et al. 2020, O’Hara et al. 2020). These EVs are produced by ESCRT-independent exosomal and autophagy-related pathways, presumably at different infection stages, based on the observation that virus release is affected by the inhibition of ceramide production and the knockout of Rab proteins, tetraspanins, or the protein GRASP65, a known regulator of secretory autophagy (Helle et al. 2020, Morris‐Love et al. 2022). Conflicting findings indicate that not all EV isolates are protected against antibody neutralization (Morris-Love et al. 2019, Handala et al. 2020). This observation could potentially stem from neutralization post cell entry, which can occur upon degradation of EV membranes within endosomal compartments, as was implicated for HEV (*Hepeviridae*). Alternatively, it indicates that infection in some cases is predominantly mediated by viral particles binding to the EV surface. The latter is still highly relevant to infection, as EVs can help both EV-enclosed and purified naked PyV virions enter cells that lack exposed sialic acids, an essential virus binding motif (Morris-Love et al. 2019, Handala et al. 2020, O’Hara et al. 2020). Likewise, in JCPyV infection, glial cells, the primary target of this virus, lack the α2,6-linked glycan lactoseries tetrasaccharide c receptor essential for naked virus binding, suggesting that EVs may play a significant role in virus entry and pathogenesis (Atkinson and Atwood 2020).

##### Single-stranded DNA viruses (ssDNA)

###### Anelloviridae

Anelloviruses have circular, negative-sense, ssDNA genomes enclosed in a nonenveloped icosahedral capsid. Their vertebrate hosts include primates, with up to 90% of humans carrying anelloviruses persistently, but without any specific disease being linked to them so far (Spandole et al. 2015). Torquetenovirus (TTV) (species *Alphatorquevirus homin* 1, 2, and 3 etc) DNA as well as intact virions have been identified in sEVs in the blood from both healthy people and patients infected with HIV, HCV, and HBV. Not all TTV strains show the same degree of EV-association, indicating that even small sequence differences may affect preferences for EV release (Martelli et al. 2018). There are speculations that EV encapsidation may confer immune evasion, reducing disease manifestation and extending viral persistence, though this remains unproven. Apart from viral DNA, EVs from anellovirus-infected cells also contain a collection of viral miRNAs, whose function is still under investigation (Vignolini et al. 2016).

###### Parvoviridae

Parvoviruses are small ssDNA viruses infecting vertebrates and invertebrates. Adeno-associated viruses (AAV) belong to a special genus of the *Parvoviridae*, and only replicate either in the presence of a helper virus such as AdV, herpesviruses, and cytomegalovirus, or under specific conditions, such as stress (Sant’Anna and Araujo 2022). AAVs are abundantly studied as possible gene-therapy and vaccine vectors (Wang et al. 2024). Most of the existing information about this family’s relation to EVs comes from studies into therapeutic vectors. Researchers showed that sEVs produced by recombinant AAV (species *Dependoparvovirus primate1*) infected cells contained viral particles, thereby enhancing protection against neutralizing antibodies (Maguire et al. 2012, Hudry et al. 2016, Li et al. 2023). EV packaging also enhances AAV transfer across the blood–brain barrier and improves penetration into complex tissues (Maguire et al. 2012, Hudry et al. 2016, György et al. 2017, Li et al. 2023). Along with virions, a collection of coding and non-coding RNAs was identified in AAV-EVs that were absent from controls; however, their function remains subject to further investigation (Li et al. 2023). Mechanistically, a viral protein produced by a frameshift, named membrane-associated accessory protein, has a possible role in AAV-EV packaging based on its enrichment on the EV surface and identified role in facilitating early virus release (Elmore et al. 2021). AAV also promotes caspase activation and cytolysis (Timpe et al. 2006), which may contribute to EV production and EV-enclosed virus release based on observations of other virus species.

#### Enveloped DNA viruses

##### Double-stranded DNA viruses (dsDNA)

###### Ascoviridae

Members of the *Ascoviridae* family are large viruses containing circular dsDNA genomes of up to 200 kb in particles of up to 400 nm in length. They rely on parasitic wasps to infect lepidopteran larvae (i.e. caterpillars). Infection halts development but increases larval lifespan, during which a specialized form of cell fragmentation resembling apoptotic blebbing occurs, which markedly increases the production of lEVs (>2 µm), as seen in Heliothis virescens ascovirus 3 h (HvAV-3 h) and Spodoptera frugiperda ascovirus 1a (SfAV-1a) (species *Ascovirus hvav3a* and *sfav1a*, respectively) infection (Yu et al. 2023b, Rudd et al. 2025). Cell fragmentation is facilitated by the synthesis of virus-encoded caspases as well as various lipid-metabolizing enzymes. The resulting lEVs in turn serve as virus assembly sites, carrying numerous virus particles (Rudd et al. 2025). Ascovirus lEVs are poorly cleared and large quantities enter the host circulation within days after infection to promote vector-borne transmission (Yu et al. 2023b). Interestingly, various types of regulated cell death pathways are modulated by ascoviruses throughout infection, suggesting a finely tuned strategy to enhance vesicle-mediated viral spread, while circumventing classical cell-death-imposed limitations to virus replication (Yu et al. 2023a).

###### Asfarviridae

*Asfarviridae* includes only one species, namely the African swine fever virus (ASFV). This virus has a linear dsDNA genome ranging from ± 170 to 194 kb, infects pigs and wild boar, and is transmitted by ticks. Produced viruses (175–215 nm) have an icosahedral capsid containing an internal lipid bilayer, and an outer lipid envelope. Using blood from ASFV-infected pigs, researchers identified sEVs positive for viral proteins (Montaner-Tarbes et al. 2019, Xu et al. 2022). These EVs appear to facilitate infection by free ASFV particles in both permissive and non-permissive cells, despite not containing viral particles or the full viral genome, only small genomic fragments (Xu et al. 2022). In cell cultures, also the release of lEVs (probably ApoBDs) has also been demonstrated during infection, which in contrast to sEVs directly contribute to cell-to-cell transmission by containing multiple virus particles that lack their own outer viral membrane (Gao et al. 2023). Uptake of these infectious EVs depends on phosphatidylserine binding to its receptor TIM4, consistent with classical ApoBDs recognition (Gao et al. 2023).

###### Baculoviridae

The *Baculoviridae* family comprises enveloped, rod-shaped viruses (up to 400 nm in length) with circular DNA ranging from 80 to 180 kb. Some genera produce two types of particles, the budded virus (BD), formed by budding at the plasma membrane, and the occlusion-derived virus (OD), which gains a distinct envelope after cell death and often encompasses multiple virus capsids (Harrison et al. 2018). As such, OD particles form a gray area between enveloped and EV-enclosed virions. Baculovirus-based expression vectors are widely used for protein expression, gene-therapy development, and VLP production for vaccines. Analysis of EVs revealed a distinct host proteomic profile upon recombinant baculovirus infection of insect cells, a finding unlikely to be explained by coisolated virions since these are expected to incorporate limited host proteins (Hausjell et al. 2023). Also for the native Autographa californica multiple nucleopolyhedrovirus (AcMNPV) (species *Alphabaculovirus aucalifornicae*), infection was shown to lead to increased production of sEVs containing altered host cargo as well as various viral proteins (Van Es et al. 2024). Together, these findings prompt a reevaluation of baculovirus-based production systems to assess or limit EV contamination and related unwanted effects. However, it should be considered that the separation of EVs and virus-derived structures remains technically demanding and often impractical, despite the development of improved methods to distinguish the two (Reiter et al. 2019, Han et al. 2023). This holds especially true for VLPs containing a lipid-bilayer, which bare many resemblances to EVs but are formed through distinct pathways as a result of their self-assembling nature (Nooraei et al. 2021).

###### Orthoherpesviridae

*Orthoherpesviridae* (180–200 nm) are notorious for causing lifelong latent infections in neurons or lymphocytes, and cause recurrent episodes of lytic virus reactivation and disease. Over 100 species are known, infecting birds, reptiles, or mammals. Modulation of EV cargo composition is observed across all genera, which is typically, but not always, joined by an increase in overall EV release (Jeon et al. 2017, 2021, Bello-Morales et al. 2018, Bergamelli et al. 2022, Ma et al. 2022, Bubak et al. 2023, Dogrammatzis et al. 2024, Niemeyer et al. 2024, Sun et al. 2024). These EVs frequently contain viral proteins and RNAs, including latent gene products (Sadeghipour and Mathias 2017, Bello-Morales et al. 2018, Cone et al. 2019, Barrett et al. 2021, Bergamelli et al. 2022, Sato et al. 2022, Niemeyer et al. 2024, Teng et al. 2024). The ability of orthoherpesviruses to affect EV release during both acute and latent infection is exemplified by the latent membrane protein 1 (LMP1), an oncogene encoded by Epstein–Barr virus (EBV) (species *Lymphocryptovirus humangamma4*), which directly interacts with CD63 to enhance its release as well as overall EV production (Hurwitz et al. 2017). A crucial role for CD63 in EV release was also shown during the lytic cycle of herpes simplex virus 1 (HSV-1) (species *Simplexvirus humanalpha1*) (Dogrammatzis et al. 2024), whereas Kaposi’s sarcoma-associated herpesvirus (KSHV) (species *Rhadinovirus humangamma8*) affects EV release via Rab27b upregulation in a GW4689-sensitive manner (Jeon et al. 2017, 2021). Although such findings point to a shared reliance on endosomal EV subsets, caution should be given to EV heterogeneity within and between settings. For example, HSV-1 and HSV-2 stimulate distinct EV subsets (Dogrammatzis et al. 2024), and HSV-1 induces a mix of endosomal and nonendosomal EVs with opposing antiviral and proviral functions (Dogrammatzis et al. 2021).

For orthoherpesviruses proviral EV functions are well documented. EVs from infected placental tissue enhanced human cytomegalovirus (HCMV) (species *Cytomegalovirus humanbeta5*) infection of neural stem cells by free virions, opening up a possible role for EVs in congenital disease (Bergamelli et al. 2022). For EBV and varicella-zoster virus (VZV) (species *Varicellovirus humanalpha3*) such proviral activity was linked to the luminal EV packaging of structural and nonstructural viral proteins that inhibit antiviral signaling pathways, whereas HSV-1 was found to shuttle essential host factors for virus replication (Sato et al. 2022, Ma et al. 2023, Niemeyer et al. 2024). Another unconventional strategy for enhancing viral spread is increasing the physical proximity between targets, as highlighted by the ability of HSV-1 glycoprotein G to promote production of EVs that stimulate neurite growth towards infected epithelial cells (Sun et al. 2024). The impact of this proviral host modulation extends beyond the initial infection, as local immune cell depletion by VZV-EVs facilitated neuroinvasion by other viruses (Niemeyer et al. 2024). Generally, the role of EVs in herpesvirus infection spread appears independent of the direct transfer of the virus. However, EV-mediated transfer of infectious virions has been shown for HSV-1, where EVs promoted infection of receptor-negative cells and enhanced resistance to neutralizing antibodies (Bello-Morales et al. 2018). Increased levels of the marker LC3 in these EVs suggest an autophagy-related packaging of HSV-1, similar to other virus families. This has not been established for other *Orthoherpesviridae*, possibly due to a focus on smaller EV subsets.

While counterintuitive, the release of EVs carrying proinflammatory or antiviral factors may also benefit *Orthoherpesviridae*. EV-mediated secretion can reduce unwanted factors in infected cells, but also produces EVs that exert antiviral effects on the surrounding. This dual role of EVs may be evolutionary beneficial to maintain a life-long host–virus equilibrium. A prime example is the CD63-dependent packaging of the antiviral sensor STING (Stimulator of Interferon Genes) in EVs, which is seen for HSV-1, HCMV, and VZV, but not HSV-2 infection (Dogrammatzis et al. 2024). EV-mediated trafficking of STING is linked to the inhibition of viral replication in uninfected cells, yet, HSV stabilizes STING, indicating this pathway is not detrimental to the virus. Similarly, EV-mediated secretion of KSHV and EBV-derived RNAs, such as EBER-1, contributes to immune evasion in infected cells while also promoting detection of viral RNA by immune cells (Baglio et al. 2016, Cone et al. 2019, Barrett et al. 2021, Burassakarn et al. 2021). Likewise, EVs can remove MHC-II molecules from infected cells to reduce antigen presentation, although in HCMV they simultaneously provide antigens for memory T cell activation (Sadeghipour and Mathias 2017). The repertoire of recognized antiviral EV cargo is still growing and varies between cell models, as indicated by studies on HSV-1 (Ma et al. 2022). Adding to this complexity, EVs may also trigger secondary antiviral cascades, such as the complement deposition triggered by endothelial KSHV-EVs, which reduced cell lysis and may reflect an ability of proinflammatory EVs to favor viral persistence (Jeon et al. 2017). The ability of inflammatory EVs to serve opposing roles in infection is even seen across species boundaries, as there is increasing evidence that even EVs coming from bacteria like *Streptococcus mutans* and *Lactobacillus rhamnosus GG*, typically rich in inflammatory motifs (e.g. lipopolysaccharides), can either promote or inhibit viral propagation by modulating cellular signaling pathways (Wang et al. 2025b, Kim et al. 2026).

Finally, EVs are also implicated in long-term herpesvirus pathology. EBV and KSHV proteins LMP1 and LMP2A, key to viral oncogenesis, are efficiently packaged into EVs (Ikeda and Longnecker 2007, Sadeghipour and Mathias 2017, Cone et al. 2019, Barrett et al. 2021). This packaging is regulated (in part) by lipids, as ceramide inhibition shifts LMP1 from tetraspanin-enriched microdomains to lipid rafts, highlighting its role in LMP1 exosomal trafficking versus oncogenic signaling (York et al. 2023). Likewise, cholesterol depletion increases LMP2A abundance and its EV secretion (Ikeda and Longnecker 2007). Latent EBV and KSHV infections, as well as Marek’s disease virus (MDV) (species *Mardivirus gallidalpha2*), a poultry pathogen, also lead to extensive packaging of viral RNA molecules and altered cellular components in EVs, which promotes tumorigenic transformation of surrounding cells (Cone et al. 2019, Barrett et al. 2021, Teng et al. 2024, Trapp-Fragnet et al. 2025). For VZV, plasma EVs display a long-term prothrombotic phenotype upon virus reactivation (up to 3 months), and still induce proinflammatory cytokines months after symptoms have resolved, suggesting a role in long-term complications such as increased stroke risk (Bubak et al. 2023).

###### Polydnaviridae

*Polydnaviridae* are viruses that have intertwined themselves with the reproductive cycle of parasitic wasps, forming a mutualistic relationship. The viral DNA is stably integrated into the wasp genome, and is passed down to their offspring via germ-line transmission to ensure virus spread in the population. Virus activation is restricted to the wasp’s ovaries, leading to localized virus production. The resulting virions are not directly transmitted between wasps, but are deposited alongside the wasp’s eggs into a secondary carrier species, in which the eggs will hatch and develop. It is in this unwilling carrier that the virus suppresses the local immune response, thereby increasing the survival rate of *polydnaviridae*-positive wasp larva and ultimately boosting virus prevalence. To modulate immunity the virus needs to enter cells and integrate its DNA for the transcription of relevant gene products. Since virus replication is not facilitated in the carrier species, it was long considered that the transcription of such products is restricted to the initial wave of cells that take up transmitted virions. However, the species *Microplitis bicoloratus bracovirus* (MbBV) was discovered to use lEVs, and specifically ApoBDs, to extend the spread of viral DNA from cell-to-cell beyond the initial wave of infection. Uptake of virus-induced lEVs was proposed to lead to the reintegration of viral DNA fragments into the genome of surrounding cells, a process that in part relies on the host homologous DNA repair system. Transcription of these viral products in turn affects transcription, translation, and cell signaling in the recipients of virus-induced EVs, leading to apoptosis and/or reduced migration, two effects that are not seen upon exposure to ApoBDs generated via nonviral triggers (Zhou et al. 2022). The release of EVs containing genomic DNA fragments during cell death, and the inadvertent incorporation of integrated viral DNA sequences upon EV uptake, poses an intriguing mechanism by which DNA viruses may maximize their impact in an otherwise nonpermissive environment.

###### Poxviridae

*Poxviridae* are large DNA viruses infecting vertebrates and arthropods, including viruses causing serious infections in humans like smallpox. A peculiarity of poxviruses, such as vaccinia virus (species *Orthopoxvirus vaccinia*), is the formation of various forms of virus particles during infection. Viral spread can be mediated by mature virions that are packaged within a single membrane in the cytosol of the infected host cells and released upon cell lysis. Cells release extracellular enveloped virions (EEV), which possess a second surrounding membrane and display a distinct viral cargo composition. To form EEV, an intermediary intracellular enveloped virus is formed surrounded by three membranes, upon wrapping of the virus in Golgi or endosomal membranes. The additional membrane of EEV poxvirus particles acts as a temporary shield to promote immune evasion during virus spread, and is lost before cellular uptake or within acidic endolysosomal compartments similar to observations for EV-enclosed viruses (Schmidt et al. 2011, Bidgood and Mercer 2015). It is not clear if EEV particles are EVs in the proper sense or virus-produced entities, but their formation relies on ESCRT-associated proteins (Bidgood and Mercer 2015). Regardless of the identity of EEV particles, bona fide EVs have been found to incorporate viral membrane proteins of the attenuated strain modified vaccina virus Ankara (Spehner and Drillien 2008), implicating dual formation of both virus and EV hybrid particles during infection. Furthermore, EVs from cattle infected with Lumpy skin disease virus (species *Capripoxvirus lumpyskinpox*) and from primary sheep cells infected with sheeppox virus (species *Capripoxvirus sheeppox*) show differential uptake of miRNAs, many of which target cellular pathways that impact the immune system (He et al. 2019, Truong et al. 2025).

##### Double-stranded DNA viruses with reverse transcribed RNA intermediates (dsDNA-RT)

###### Hepadnaviridae

Hepadnaviruses are small (<50 nm) enveloped viruses with a partially dsDNA/ssDNA genome. These viruses target mammals, reptiles, frogs, and fish, causing transient or persistent infection. The most well-known member of this family is HBV (species *Orthohepadnavirus hominoidei*), which during infection produces infectious virions, also known as Dane particles, as well as abundant genome-free spherical and filamentous subviral particles (Magnius et al. 2020). The classification of HBV subviral particles in the spectrum between virions and EVs remains ill-defined, highlighting the need for more precise definitions within this evolving field. Likewise, the functions of these subviral particles are not fully solved, although an antibody-decoy role has been proposed. Aside from the possible crossover with HBV subviral particles, evidence suggests a close relationship between EV biogenesis machinery and the HBV life cycle, with possible mutual consequences for both EV and virus release. HBV increases sEV production in both cell lines and patient samples, which in part is believed to be mediated by increased expression of EV biogenesis proteins (Kouwaki et al. 2016, Kapoor et al. 2017, Yang et al. 2017b, Todorova et al. 2023, Wu et al. 2023). Within infected cells, CD63 colocalizes with HBV capsids, and regulates the secretion of viral envelope proteins (Ninomiya et al. 2021). Its depletion reduces the infectivity of EV/virus mixtures containing equivalent amounts of genome copies, although whether this is caused by altered virus or EV composition is unclear (Ninomiya et al. 2021). A packaging of HBV virions inside EVs has been observed, which could be reduced using both GW4869 and U18666A, while the abundance of viral DNA in non-EV density fractions remains unaffected (Wu et al. 2023). The release of HBV-carrying EVs is similarly affected by the host factors Alix and syntenin, which together support exosomal virus packaging (Wu et al. 2023). The resulting EVs constitute an alternative transmission pathway, as density gradient centrifugation-purified EVs containing HBV virions were able to enter cells and establish infection (Wu et al. 2023). Alternatively, EV-mediated transfer of virions can induce NK cell dysfunction, thereby interfering with immune cell activation (Yang et al. 2017b). Alongside intact virions, EVs contain altered endogenous proteins and miRNAs, as well as HBV–DNA, HBV–RNA, and proteins that can contribute to infection progression or disease (Kouwaki et al. 2016, Kapoor et al. 2017, Yang et al. 2017b, Todorova et al. 2023, Wu et al. 2023). Notable examples include an HBV-encoded miRNA, named HBV-miR-3, known to regulate viral replication and promote persistent infection (Yang et al. 2017a). At the same time, enclosed host miRNAs enable HBV-EVs to lower the antiviral activity of macrophages by altering the levels of key immune regulators, including pro-inflammatory interleukin-12 (IL-12) (Kouwaki et al. 2016, Hurwitz et al. 2017, Zhang et al. 2024c). Likewise, EV-enclosed proteasome proteins are linked to inhibited cytokine secretion (Jia et al. 2017). It is worthy to note that by incorporating viral glycoproteins HBV increases the uptake of virus-induced EVs by susceptible cells, thereby potentially enhancing the potency of pathological EVs (Wu et al. 2023). On the other hand, HBV-target cells can also take up EVs released by nonpermissive cells, a phenomenon that plays an important role in IFN-α-mediated restriction of virus replication (Li et al. 2013).

### DNA/RNA viruses of mixed taxonomical origin infecting prokaryotes

#### Bacteriophages

Bacteriophages is the collective term for a large number of virus families infecting bacteria. Most available bacteriophage sequences (>90%) belong to species with a dsDNA genome and a typical tailed morphology; however, bacteriophages exist with different virion shapes and nucleic acid genomes (Zrelovs et al. 2020). Given recent and ongoing taxonomic revisions (Turner et al. 2023), we discuss this group as a whole. In recent years, bacteriophages have gained significant interest as an alternative “weapon” against antibiotic-resistant bacteria, in addition to their essential roles in evolution, carbon and nutrient cycling, population diversity, and beyond. Bacteriophages infect both Gram-positive and Gram-negative bacteria, which release EV subsets containing cytoplasmic and/or outer membrane-derived lipid bilayers, collectively termed BEVs. BEV biology during phage infections remains poorly understood but most studies report an increase in BEV release (reviewed in Meidaninikjeh et al. 2024), driven primarily by phage-encoded holin–endolysin systems that damage the cell wall or membrane (Turnbull et al. 2016, Liu et al. 2022b). These viral factors are often linked to a specific form of cell death, referred to as “explosive cell lysis” or “bubbling cell death” depending on the bacterial cell wall. In turn, this cell death process, which can also be triggered by various nonviral stressors, is considered a major contributor to BEV production in both gram-positive and negative contexts (Turnbull et al. 2016, Toyofuku et al. 2017, Mandal et al. 2021). Yet, there are also indications for (holin–endolysin promoted) virus–BEV interactions that precede or happen independent of death of the bacterium (Mandal et al. 2021, Liu et al. 2022a, 2022b). In fact, in *Lactococcus lactis* membrane packaging of typically nonenveloped phages was proposed to represent a nonlytic virus release strategy (Liu et al. 2022a). At the moment, membrane-enclosed viral particles have been identified with (T)EM imaging of phage-harboring *L. lactis* (phage proPhi1) and *Bacillus subtilis* (phage PSBX) cultures, however, viral components have not yet been conclusively demonstrated in purified BEV isolates (Toyofuku et al. 2017, Liu et al. 2022b). Ongoing investigations aim to clarify whether phages are packaged into BEVs via lytic or nonlytic routes and whether such BEVs can transmit infection.

While proviral roles of BEV are still debated, increased BEV production in response to virus exposure has been established as an efficient antiviral stress response or defense strategy within bacterial communities. Numerous studies show that BEVs act as decoys for bacteriophage binding, as was for example proposed for *Escherichia coli* cultures exposed to T4 phages (Meidaninikjeh et al. 2024, Xuan et al. 2024). Decoy efficiency depends on the abundance of phage receptors on different BEV subsets, whether released spontaneously or through cell death (Stephan et al. 2020). Work on OMV-mediated protection of *Pseudomonas aeruginosa* highlights that additional phage entry requirements likely determine the ability of BEVs to mediate complete virus neutralization, which for example can be caused by the triggering of viral genome injection into the BEV lumen (Stephan et al. 2020, Augustyniak et al. 2022). For Gram-positive bacteria, it should be considered that peptidoglycan cell wall components are typically not incorporated into BEVs, meaning that many primary virus attachment factors are not present to aid in BEV decoy activity. Yet, the ability of Gram-positive BEV release to modulate virus entry is emphasized by the finding that BEVs from the Gram-positive bacterium *Bacillus subtilis* could transiently render resistant cells susceptible to infection (Tzipilevich et al. 2017), indicating that BEVs may also promote infection by transferring viral receptors or nucleic acids that encode them. However, this has not been confirmed in other settings (Augustyniak et al. 2022, Xuan et al. 2024). The other way around, bacteriophages can also affect the uptake of BEV even across kingdoms. Binding of the filamentous phage Pf4 to the outside of BEVs has clearly been demonstrated, altering BEV internalization and subsequent activation of human macrophages (Pennetzdorfer et al. 2023). By effectively altering cross-kingdom EV communication, Pf4 is therefore suggested to facilitate immune evasion of its host (*P. aeruginosa*).

#### Archaeal viruses

While bacteria often claim the spotlight, a second important class of prokaryotes to consider are the archaea. Although archaea appear similar to bacteria under the microscope, they are molecularly and genetically very different. One key difference (for the purposes of this review) lies in their membrane composition: archaea possess ether-linked lipids, whereas bacteria have ester-linked lipids, which contributes to the remarkable ability of archaea to survive in extreme environments. Their membranes are also more similar to those of eukaryotes, often consisting of a single lipid layer without a surrounding cell wall, and specific archaeal lineages support EV production through a molecular machinery resembling that of eukaryotic cells (Soler et al. 2008, Liu et al. 2021b). Archaeal viruses are largely understudied and often possess completely different features compared to eukaryotic/bacterial viruses. In the context of EVs, it has been proposed that the virus Sulfolobus tengchongensis spindle-shaped virus 2 (STSV2) (unclassified *Fuselloviridae*) influences ESCRT proteins, leading to cell cycle arrest and cell gigantism, and thereby potentially also affects the budding of vesicle-like structures regulated by these same proteins (Liu et al. 2021a). Recent analysis of the EV release by *Methanobrevibacter smithii*, part of the gut microflora, has revealed the preferential packaging of excised proviral DNA of Methanobrevibacter smithii tailed virus 1 (MSTV1) (species *Manusuvirus methanobrevibacteri*) in EVs (Baquero et al. 2025). Similarly, vesicle-like structures (resembling EVs) from *Thermococcus nautilus* were shown to contain (defective) viral genomes harbored in the form of plasmids (Soler et al. 2011, Gaudin et al. 2014), opening up future studies into viral–EV interactions in this area. Currently, research in archaea biology is challenging our understanding of what defines a virus, a plasmid, and an EV. A striking example is pR1SE, a plasmid identified in Antarctic haloarchaeon, that uses EVs to move between cells. The membranes of these EVs are enriched with proteins encoded by the plasmid itself, mimicking the packaging of viral genomes by capsid proteins, a finding typically used to distinguish between plasmid and virus spread (Erdmann et al. 2017). This hybrid behavior has led researchers to propose a new biological entity, termed “plasmidions,” representing plasmids that adopt virus-like strategies for dissemination (Forterre et al. 2017). The other way around, EV-mediated virus transfer may contribute to the misidentification of archaeal viruses as plasmids.

## Summary and outlook

This review has summarized the current knowledge across the full range of viruses known to directly or indirectly engage EVs during their biological cycle. In doing so, it provides researchers a platform to systematically explore the state-of-the-art in areas that they may otherwise have not considered. We believe that by comparing findings across diverse infection models, lessons can be learned that advance the knowledge about EVs and infection biology within and across disciplines, as many recurring features are observed across these (arbitrary) boundaries (Figs 1 –3). While EV–virus interactions are being characterized across an expanding range of biological contexts, a systematic assessment of current findings indicates that several areas remain un- or underexplored, most notably fungal and plant viruses. Likewise, it remains an open question to what extent findings will parallel those for virus-like pathogens, such as ambiviruses, mitoviruses, viroid-like, or DNA/RNA satellite pathogens (Navarro and Turina 2024). Ongoing developments in the understanding of EV release by different organisms potentiate rapid steps to close these and other remaining knowledge gaps in the future. In particular, improved technical practices regarding the study of EV–virus mixtures, along with more clear definitions demarking the gray area between EVs and viral structures, are expected to facilitate future progression in this field.

Ultimately, increased understanding and recognition of EV release and function is important for many aspects of virology research. For too long, the release of EVs has been overlooked, with possible consequences on matters such as the quality and properties of virus stocks, interpretation of neutralization assays, the study of excreted viral virulence factors, and more. As these insights accumulate, their relevance is extending beyond basic research and into translational applications. Knowing the role of EVs in viral pathogenesis helps guide the optimal design of antivirals and vaccine strategies, as well as predict or understand residual pathologies in individuals receiving treatment. These efforts are aided by the selective profiling of EVs in body fluids, which forms the foundation for EV-based diagnostics. Moreover, current data from the AdV and AAV field highlights the beneficial properties of using EV-enclosed instead of nonenveloped therapeutic viruses. Improved understanding of the packaging of viral cargo into EVs and the functional properties of different EV subsets can lead to a directed development of such viral vectors, as well as efforts to use EVs as carriers of viral antigens in vaccine development. Also in agriculture and aquaculture, EVs may be used to enhance antiviral responses, by, for example, improving the stability and uptake of antiviral siRNAs in spray–induced gene silencing (He et al. 2024) or by acting as natural vaccines, as shown for BEVs in fish (Escribano et al. 2024). We anticipate that many more applications will emerge based on our increasing knowledge on EV–virus interactions. These include new quality controls for disinfection protocols based on the increased environmental resistance observed for EV-associated viruses, or the use of EVs as intervention targets to reduce direct pathological effects or those indirectly caused by cross-kingdom exchange of viral EVs. The more researchers are able to extrapolate from and build upon advances across different areas of EV–virus research, the more rapidly such applications may become a reality.
